# Boron subphthalocyanine complexes for CO_2_ electroreduction: molecular design and catalytic insights

**DOI:** 10.1039/d5ya00136f

**Published:** 2025-09-05

**Authors:** Farzaneh Yari, Simon Offenthaler, Sankit Vala, Dominik Krisch, Markus Scharber, Wolfgang Schöfberger

**Affiliations:** a Institute of Organic Chemistry, Johannes Kepler University Linz Altenberger Staße 69 4040 Linz Austria wolfgang.schoefberger@jku.at; b Institute of Physical Chemistry and Linz Institute of Organic Solar Cells (LIOS) Johannes Kepler University Linz (JKU) Altenberger Straße 69 4040 Linz Austria

## Abstract

This study presents molecular boron subphthalocyanine complex precursors ((Cl-B-SubPc) 1 and (Cl-B-SubPc-OC_12_H_23_) 2) designed for efficient CO_2_ reduction. The resulting heterogeneous catalysts exhibit remarkable total faradaic efficiencies of up to 98%, integrated into practical cell assemblies. Optimizations encompass not only catalyst design but also operational conditions, facilitating prolonged CO_2_ electrolysis across various current densities. Varied C_1_-, C_2_-, and C_3_-product yields are observed at different reductive potentials, with electrocatalysis experiments conducted up to 200 mA cm^−2^. Comparative electrochemical analyses across H-cell and zero-gap cell electrolyzers show the potential for industrial scale-up. Mechanistic elucidation *via in situ* UV-vis spectroelectrochemistry, DFT calculations, and ESR spectroscopy demonstrates the involvement of boron N–C sites, initiating radical formation and utilizing boron's Lewis acid behavior in CO_2_ capture, followed by proton-coupled electron transfer. Overall, the study underscores the transformative potential of boron subphthalocyanine systems in advancing CO_2_ utilization technologies.

## Abstract 2

Boron subphthalocyanine precursors enable efficient CO_2_ reduction, with optimized design and conditions supporting stable electrolysis and tunable product yields at high current densities.

## Graphical abstract

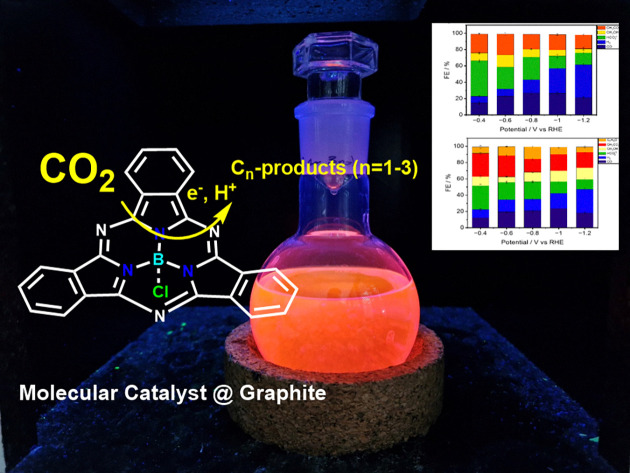

## Introduction

The electrochemical reduction of carbon dioxide (CO_2_) to value-added chemicals offers a promising route toward carbon neutrality by enabling the utilization of C_1_ feedstocks under mild conditions.^1–6^ Among the range of products accessible *via* the CO_2_ reduction reaction (CO_2_RR)—including CO,^7–10^ formate,^11,12^ methane,^13,14^ methanol,^15,16^ ethylene,^17–19^ and ethanol^20^—the selective production of CO is particularly attractive due to its use in syngas and its relatively low energy barrier. While considerable progress has been made using transition metal-based catalysts, such as Cu, Pd, Au, and Ag, these materials suffer from issues including high cost, limited availability, and poor long-term stability, impeding their practical applications. This has prompted growing interest in the development of noble metal-free electrocatalysts with high activity, selectivity, and stability.^21–25^

In this context, molecular catalysts—particularly those based on non-transition metal elements—have emerged as promising candidates due to their tunable coordination environments and well-defined active sites.^26,27^ Among them, boron–nitrogen (B–N) coordination compounds are especially intriguing for the CO_2_RR.^28,29^ These systems benefit from unique electronic properties arising from the B–N interaction: boron acts as an electron-deficient Lewis acid, while nitrogen serves as an electron-rich donor, allowing for cooperative activation of CO_2_. Furthermore, the incorporation of B–N motifs into conjugated macrocycles, such as subphthalocyanines or related frameworks, offers the possibility of electronic delocalization, redox modulation, and geometric control—features highly advantageous for selective electrocatalysis.

Recent advances in B,N co-doped carbon-based materials have demonstrated significant CO_2_RR activity, underscoring the catalytic relevance of B–N synergy.^28–35^ However, the use of discrete molecular B–N coordination complexes as electrocatalysts remains comparatively underexplored. These molecular systems offer the benefit of well-defined structures, facilitating mechanistic insights that are challenging to extract from heterogeneous catalysts. Moreover, the development of B–N coordination complexes that operate across a wide pH range—including acidic media—would address limitations associated with carbonate formation and HER suppression, especially under neutral or alkaline conditions.^36^

Despite encouraging preliminary findings, key questions remain regarding the mechanisms of B–N activation, the role of molecular geometry, and the stability of such complexes under electrochemical conditions. Thus, there is a compelling need to design and investigate boron–nitrogen coordination compounds as molecular electrocatalysts for the CO_2_RR, with a particular focus on structure–activity relationships, pH tolerance, and electron transfer characteristics.

We herein investigate the electrocatalytic performance of molecular boron subphthalocyanines for CO_2_ reduction. By leveraging the properties of the central boron atom in a conserved coordination sphere, we develop a catalyst ink that exhibits high selectivity and activity for the conversion of CO_2_.

## Experimental

All chemicals were purchased from TCI or Sigma-Aldrich and used without further purification. Solvents for the NMR were purchased from Euriso-Top. TLC was performed on Macherey-Nagel silica gel 60 (0.20 mm) with the fluorescent indicator UV254 on aluminum plates and on Merck aluminum oxide 60 (0.20 mm) with the fluorescent indicator UV254 on aluminum plates. For chromatography, silica-gel columns were prepared using silica-gel 60 (0.070–0.20 mesh) from Grace. Proton (^1^H-NMR) and carbon (^13^C-NMR) spectra were recorded on a Bruker Ascend 700 MHz Advance III NMR spectrometer equipped with a cryoprobe, a DRX 500 MHz spectrometer and a Bruker Avance III 300 MHz NMR spectrometer. Phosphorus (^11^B-NMR) spectra were recorded on a Bruker Avance III 500 MHz spectrometer equipped with a cryoprobe (TXI) at 160.42 MHz. The chemical shifts are given in parts per million (ppm) on the delta scale (*δ*) and are referred to the used deuterated solvent for ^1^H-NMR. High resolution mass spectra were obtained using an Agilent 6520 Q-TOF mass spectrometer with an ESI source and an Agilent G1607A coaxial sprayer or a Thermo Fisher Scientific LTQ Orbitrap XL with an Ion Max API Source. UV-vis absorption spectra were collected on a Varian Cary 300 Bio spectrophotometer from 200 to 900 nm.

### Materials synthesis

#### General procedure for the synthesis of compounds 1 and 2

Phthalonitrile derivatives (0.50–0.74 g, 1.6–3.9 mmol) were dissolved in mesitylene (20–30 mL) under an argon atmosphere. Boron trichloride (7.5–9.5 mL, 1.0 M in CH_2_Cl_2_) was added dropwise, and the mixture was refluxed for 2 h. Reaction progress was monitored by TLC (*n*-heptane/CH_2_Cl_2_, 1 : 3). Upon completion, the solvent was removed under reduced pressure, and the crude product was dried in a desiccator. The solid was washed sequentially with toluene and methanol, then purified *via* silica gel column chromatography (heptane : ethyl acetate, 3 : 1, *R*_f_ = 0.68) to afford the desired BSubPc as a purple solid. Yield: 73–85%.

### Materials characterization

A comprehensive characterization was conducted using NMR, UV-Vis, and XPS techniques, verifying the chemical structures of the catalysts. (Cl-B-SubPc) 1 and (Cl-B-SubPc-OC_12_H_23_) 2 were deposited on carbon paper through drop casting using a methanol mixture and subjected to X-ray photoelectron spectroscopy (XPS) analysis both before and after electroreduction. XPS was performed by using a Theta Probe (Thermo Fisher, UK) using monochromatic Al Kα X-rays (*hν* = 1486.6 eV), with a spot size of 400 microns and a photoelectron take-off angle of 90° with respect to the surface plane. The binding energies were corrected using the C 1s peak at BE = 284.6 eV that arises from adventitious hydrocarbon. The catalytic loading determined by weighing before and after spray-coating was 0.2 mg cm^−2^. Note that, for XPS characterization, the GDLs were prepared similarly, except without the addition of Nafion^TM^ N117 (Chemours) in order to avoid suppression of other elements' intensity by mainly carbon and oxygen.

### Testing electrochemical activity for the CO_2_RR

The electrochemical properties of the electrocatalysts were systematically examined through both homogeneous and heterogeneous approaches. Homogeneous electro-characterization was specifically employed to assess catalyst responsiveness to CO_2_ and involved cyclic voltammetry measurements. Glassy carbon served as the working electrode, a Pt wire as the counter electrode and a AgCl coated Ag wire as electrode pseudo-reference electrode in 10 mL of DCM with 0.1 M TBAP as the supporting electrolyte, employing a scan rate of 30 mV s^−1^. Conversely, heterogeneous measurements were conducted to elucidate electrochemical phenomena that could be encountered in scaled-up applications. All heterogeneous electrochemical measurements, except for zero-gap cell experiments where noted, were carried out in an H-type cell, where compartments were separated by a Nafion membrane. Before measurements, the electrolyte solution (0.1 M CsHCO_3_) was purged with CO_2_ for one hour at a flow rate of 50 mL min^−1^ and then bubbled continuously with CO_2_ at 10 mL min^−1^ during the test until the pH of the saturated solution reached 6.8 after 1 h. 0.1 M CsHCO_3_ was chosen as the electrolyte, as H_2_ formation was lowest in this case. Boron subphthalocyanine chlorides (Cl-B-SubPc) 1 and (Cl-B-SubPc-OC_12_H_23_) 2 were physisorbed on carbon paper as a supportive electrode with an effective loading of 0.2 mg cm^−2^ and tested in a three-electrode configuration with the catalyst loaded cathodes as working electrodes, Ag/AgCl/1 M KCl as the reference and a platinum wire as the counter electrode. Cyclic voltammetry (CV) measurements under heterogeneous conditions were conducted to study the electrocatalytic efficiency towards the CO_2_ reduction reaction (e-CO_2_RR) in an aqueous electrolyte solution. They were performed under argon and CO_2_ in 0.1 M CsHCO_3_ (pH 6.8) electrolyte solution. Potentiostatic chronoamperometry (CA) was conducted to measure the consumed electrons during electrosynthesis in coulombs by integration of the current over time. Throughout the electrolysis, CO_2_ gas was introduced into the cathodic compartment of the H-cell at a flow rate of 10 mL min^−1^ to maintain a CO_2_-saturated environment. The voltage on the working electrode was incrementally adjusted, ranging from −0.4 to −1.2 V *vs.* RHE, and held constant for one hour with stirring at each potential to record the corresponding chronoamperometric curve. The electrochemical active surface area (ECSA, cm^2^) was calculated using double-layer capacitance, *C*_DL_, which was measured by conducting CV within a 200 mV window centered at −0.02 V *vs.* RHE for (Cl-B-SubPc) 1 and at 0.1 V *vs.* RHE for (Cl-B-SubPc-OC_12_H_23_) 2. All potentials were eventually transformed to the reversible hydrogen electrode reference using the following relationship:*E*_*vs*. RHE_ = *E*_*vs*. Ag/AgCl_ + 0.209 V + 0.0592 V × pH

The different current densities (*i*_c_, mA cm^−2^) were plotted as a function of the scan rate (*v*, mV s^−1^) with a slope equal to *C*_DL_ (μF cm^−2^). The electrochemically active surface area (ECSA) is estimated by relating the double-layer capacitance (*C*_DL_, μF) to that of a smooth planar surface (*C*_REF_, typically assumed as 40 μF cm^−2^), using the following equations:
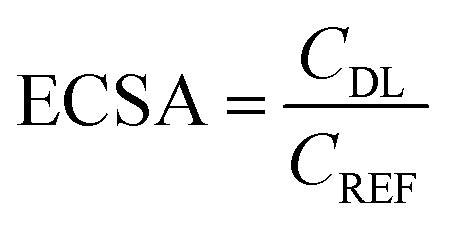
*C*_DL_ = *C*_DL_ × *S* (*S* is the surface area of the electrode, cm^2^)

For the zero-gap cell experiments related to CO_2_ electroreduction, the cathode gas diffusion electrode (GDE), coated with the catalyst (geometric active area of 9 cm^2^ with a catalyst loading of 1 mg cm^−2^), was separated from the anode by an anion exchange membrane (PiperION A40-HCO_3_). The membrane was conditioned overnight in 1 M KOH and washed with Milli-Q water before electrolysis. The employed anode featured a loading of 1 mg cm^−2^ IrO_2_. 0.1 M CsOH was employed as the anolyte and circulated through the anode flow channels, while gaseous CO_2_ was fed into the cell on the cathode side. Utilizing a temperature-controlled humidifier, the relative humidity of the CO_2_ gas was adjusted based on the applied current density. For each CO_2_ reduction experiment, fresh electrolyte was prepared, and it was circulated through the electrochemical cell using peristaltic pumps at a rate of 50 mL min^−1^. An automatic mass flow controller maintained the flow of the input CO_2_ (99.99%) at 100 sccm throughout each experiment.

### Theoretical investigations *via* DFT

All quantum chemical calculations were performed using the Gaussian 16 software package.^37^ Geometry optimizations of the chloro-boron subphthalocyanine (Cl-B-SubPc, complex 1) were carried out using density functional theory (DFT) with the B3LYP hybrid functional,^38^ combined with the 6-311G basis set (valence triple-ζ quality)^39^ for all atoms (B, Cl, O, N, C, and H). Grimme's D3 dispersion correction with Becke–Johnson damping (D3BJ) was applied to account for non-covalent interactions.^40^ Both singlet (low-spin) and triplet (high-spin) states were optimized to identify the energetically favored ground state. Frequency calculations at the same level of theory confirmed that the optimized structures correspond to true local minima (no imaginary frequencies). Implicit solvation effects were included using the integral equation formalism of the polarized continuum model (IEF-PCM) with acetonitrile (CH_3_CN) as the solvent to better simulate experimental electrochemical conditions. Excited-state properties were evaluated using time-dependent DFT (TD-DFT) at the B3LYP-D3BJ/6-311G level on the optimized ground-state geometries. Molecular orbitals, HOMO–LUMO energies, and electronic transitions were analyzed to gain insight into the electronic structure relevant to CO_2_ reduction catalysis. Additional computational data, including orbital visualizations and excitation parameters, are provided in the SI.

## Results and discussion

**Fig. 1:**
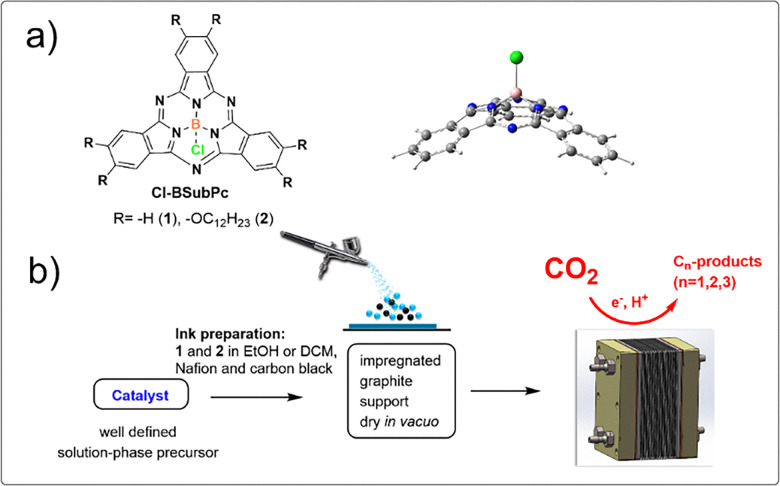
(a) Chemical structures of boron subphthalocyanine chlorides (Cl-B-SubPc) 1 and (Cl-B-SubPc-OC_12_H_23_) 2(Cl-B-SubPc) 1 and (Cl-B-SubPc-OC_12_H_23_) 2, and DFT-optimized geometry of (Cl-B-SubPc) 1 and (b) the preparation of catalyst inks for impregnation on a graphite support material (carbon paper or gas diffusion electrodes) for electrocatalytic CO_2_ conversion in H-cell and zero-gap cell electrolysers.

**Fig. 2:**
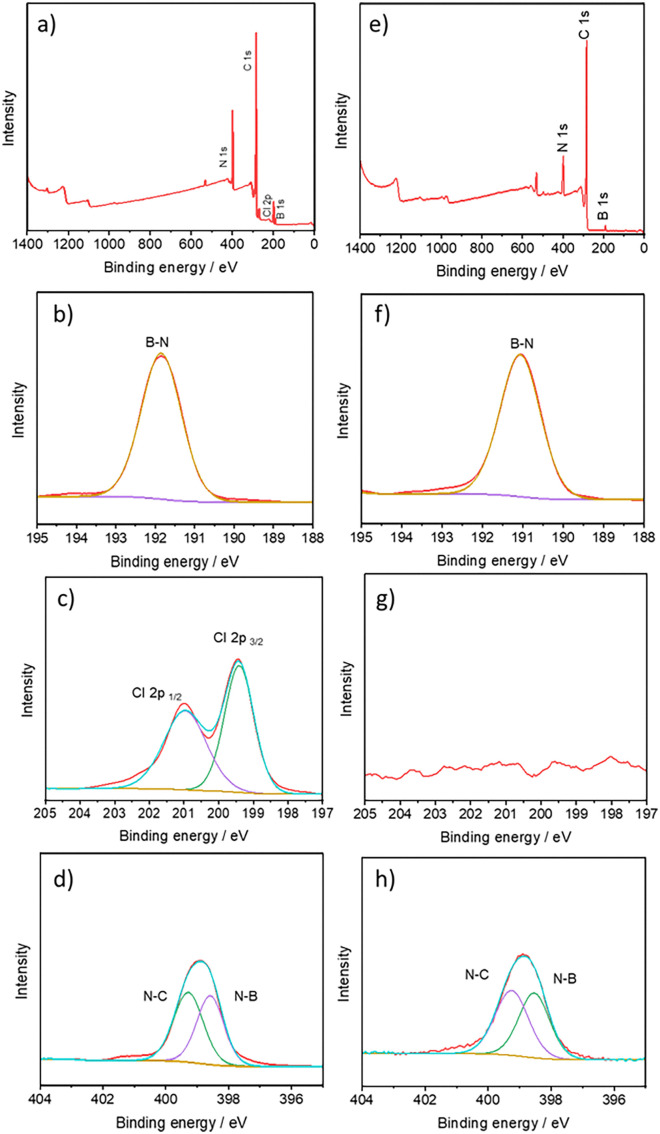
X-ray photoelectron spectra of Cl-B-SubPc 1 before (a–d) and after electrocatalysis (e–h).

Two boron subphthalocyanine chlorides (Cl-B-SubPc) 1 and (Cl-B-SubPc-OC_12_H_23_) 2 serve as the molecular platforms for preparing the heterogeneous catalyst material. Boron SubPcs are a class of cone-shaped macrocycles formed by three isoindole units connected by imine bridges that surround a central boron atom with an axial substituent (see Fig. 1a). Since the angle between the pyrrole nitrogens and the boron atom deviates from both the ideal 109.5° (sp^3^) and 120° (sp^2^) geometries, the hybridization of the boron center is likely intermediate between sp^2^ and sp^3^. This geometry gives rise to a bowl-shaped structure, a 14-π electron delocalized system, and overall *C*_3v_ symmetry. The highest occupied molecular orbital (HOMO) and the lowest unoccupied molecular orbital (LUMO) are delocalized across the macrocyclic ligand, indicating that the first oxidation and reduction events in boron subphthalocyanine (BSubPc) are macrocycle-centered and involve one-electron processes.^41^ Historically, the one-electron oxidation of BSubPcs has been reported as typically irreversible, whereas the reversibility of the one-electron reduction depends on the nature of peripheral substituents.^42,43^ Recent studies have shown that Cl-B-SubPc 1 can undergo reversible oxidation at elevated temperatures^44^ in dichloroethane (C_2_H_4_Cl_2_) solutions containing [*n*Bu_4_N][B(C_6_F_5_)_4_] as the supporting electrolyte. Extending the potential window—both anodically and cathodically—can access additional redox processes. Consequently, further oxidation and reduction events have been observed in BSubPcs under such conditions.^42^ The synthesis of SubPcs typically involves the cyclization of phthalonitriles in the presence of boron(iii) salts. While other, larger metal ions facilitate the formation of four-membered macrocycles (phthalocyanines), the small boron core acts as a template for three-membered rings. The atomic radius of boron slightly exceeds the size of the binding pocket of the SubPc macrocycle, forcing it to adapt a cone shape. The central boron ion coordinates to three pyrrole nitrogens and one axial ligand on the convex side of the macrocycle. Next, inks were prepared by combining 2 mg of the electrocatalysts (Cl-B-SubPc) 1 and (Cl-B-SubPc-OC_12_H_23_) 2 with 2 mg carbon black in 2 mL of methanol. To enhance the adhesion of the ink, 20 μL of a 5 wt% Nafion 117 solution (Sigma-Aldrich) was incorporated as a binder. The resulting catalytic inks underwent sonication for 30 minutes. They were subsequently sprayed onto a 1 × 1 cm^2^ surface of carbon paper (TGP-H-60, Thermo Scientific) for H-cell characterization. In contrast, for zero-gap cell measurements, a gas diffusion layer (GDE, CeTech) was applied (W1S1011-365 μm) and fully dried under vacuum overnight. X-ray photoelectron spectroscopy (XPS) of compounds 1 and 2 affords elemental ratios of B 1s, Cl 2p_1/2_, Cl 2p_3/2_, N 1s, and C 1s of 1 : 1 : 6 : 24 and 1 : 1 : 6 : 72, respectively, consistent with the presence of molecularly adsorbed species at the surface. High-resolution spectra of B 1s and Cl 2p for (Cl-B-SubPc) 1 and (Cl-B-SubPc-OC_12_H_23_) 2 at room temperature exhibit binding energies of 192.0 eV (B 1s), 201.2 eV (Cl 2p_1/2_), and 199.4 eV (Cl 2p_3/2_), characteristic of axially coordinated chloride ligation (Fig. 1b and c). The N 1s region displays two distinct features: a peak at 399.5 eV, assigned to the iminic =N– moiety, and a peak at 398.7 eV, attributable to the central B–N coordination sphere (Fig. 2d). Post-electrocatalytic XPS analysis (Fig. 2e–h) reveals complete disappearance of the Cl 2p signal, accompanied by a shift in the B 1s peak from 191.9 to 191.0 eV, indicative of chloride dissociation from the axial site. In contrast, the N 1s envelope remains unchanged in both position and line shape, underscoring the stability of the macrocyclic coordination environment.

Boron subphthalocyanines display characteristic UV/vis absorption bands, notably a strong Q-band associated with π–π* transitions of the conjugated macrocycle, and a B-band (or Soret band) in the UV region (Fig. S9). For (Cl-B-SubPc) 1, the absorption maxima were observed at 269 nm and 306 nm (B-band) and at 562 nm (Q-band). In the case of (Cl-B-SubPc-OC_12_H_23_) 2, the absorption maxima appear at slightly higher wavelengths—271 nm, 307 nm (B-band), and 571 nm (Q-band). The red shift of approximately 9 nm in the Q-band for the alkoxy-substituted derivative reflects increased electron-donating effects and extended conjugation introduced by the peripheral alkoxy groups. This substitution influences the electronic distribution within the macrocycle, lowering the energy gap between the HOMO and LUMO, and thereby shifting the Q-band absorption to longer wavelengths.

**Fig. 3:**
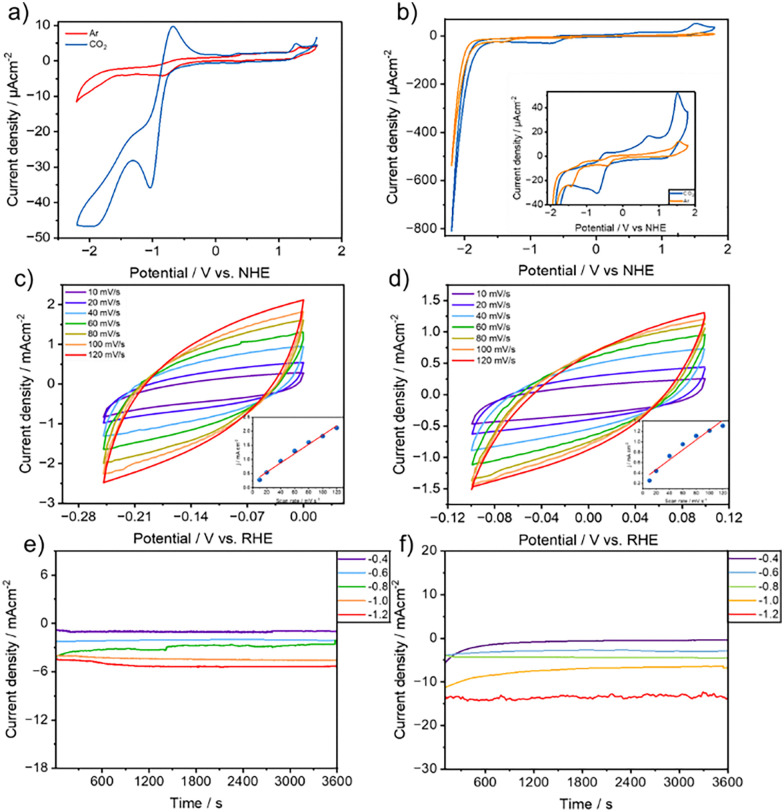
(a) Comparison of the cyclic voltammograms of (Cl-B-SubPc) 1 dissolved in DCM under argon and CO_2_ containing 0.1 M TBAP as the supporting electrolyte with glassy carbon as the working electrode, platinum wire as the counter electrode, and nonaqueous pseudo-Ag/AgCl as the reference electrode with a scan rate of 30 mV s^−1^. (b) Comparison of the cyclic voltammograms of (Cl-B-SubPc-OC_12_H_23_) 2 dissolved in DCM under argon and CO_2_ containing 0.1 M TBAP as the supporting electrolyte with glassy carbon as the working electrode, platinum wire as the counter electrode and nonaqueous pseudo-Ag/AgCl as the reference electrode with a scan rate of 30 mV s^−1^. (c) CV curves of (Cl-B-SubPc) 1 at different sweep rates of 10–120 mV s^−1^ from −0.25 to 0.0 V in 0.1 M CsHCO_3_; inset: a linear plot of capacitive current *versus* the scan rate (mV s^−1^). (d) CV curves of (Cl-B-SubPc-OC_12_H_23_) 2 at different sweep rates of 10–120 mV s^−1^ from −0.1 to 0.1 V in 0.1 M CsHCO_3_; inset: a linear plot of capacitive current *versus* the scan rate (mV). (e) Cell current *vs.* time plot at different half-cell potentials *vs.* RHE of 1. (f) Cell current *vs.* time plot at different half-cell potentials *vs.* RHE of (Cl-B-SubPc-OC_12_H_23_) 2.

The electrochemical properties of 1 mM (Cl-B-SubPc) 1 and (Cl-B-SubPc-OC_12_H_23_) 2 in DCM were examined using cyclic voltammetry (CV) under both argon and CO_2_ atmospheres. The experiments utilized a glassy carbon electrode as the working electrode, with 0.1 M TBAP serving as the supporting electrolyte, and a scan rate of 30 mV s^−1^. As illustrated in Fig. 1a (orange curve), distinct quasi-reversible one-electron redox peaks were detected at −0.8 V *vs.* NHE, indicative of ligand-centered electroreduction. Under a CO_2_ atmosphere, the same reversible redox peak was observed at this potential, but it was accompanied by a significant increase in current (Fig. 3a, blue curve), highlighting the catalytic activity of (Cl-B-SubPc) 1 for CO_2_ reduction. (Cl-B-SubPc-OC_12_H_23_) 2 exhibits a quasi-reversible one-electron reduction at −0.94 V *vs.* NHE and a quasi-reversible one-electron reduction at −1.42 V *vs.* NHE, which are similar to (Cl-B-SubPc) 1. Linear sweep voltammetry was conducted in a 0.1 M CsHCO_3_ aqueous electrolyte, with CsHCO_3_ chosen for its alleviating effect on parasitic H_2_ formation. Initially, Ar gas (99.99%) was purged for 15 minutes through the CsHCO_3_ solution to eliminate air. Subsequently, experiments were carried out in a 0.1 M CsHCO_3_ solution saturated with gaseous CO_2_ (99.99%) at a flow rate of 10 mL min^−1^ until the pH of the saturated solution reached 6.8 (∼60 minutes) (Fig. S14 and S15). The experiments were performed at room temperature. (Cl-B-SubPc) 1 and (Cl-B-SubPc-OC_12_H_23_) 2 both demonstrated significantly higher current densities in a CO_2_-saturated electrolyte compared to an Ar-saturated environment (Fig. S14 and S15), clearly indicating their electrocatalytic activity toward CO_2_ reduction.

This enhanced current response under CO_2_ suggests that both catalysts actively participate in the electrochemical conversion of CO_2_, likely facilitating key reduction steps that are absent in the inert Ar atmosphere.

These observations highlight the potential of (Cl-B-SubPc) 1 and (Cl-B-SubPc-OC_12_H_23_) 2 as promising candidates for CO_2_ electroreduction applications. In the aqueous medium, the cell current observed in the electroreduction of CO_2_ (e-CO_2_R) was attributed not only to the e-CO_2_RR but also to H_2_ gas generation, making it challenging to distinguish from the linear sweep voltammetry (LSV) whether the observed peak can be accounted for the e-CO_2_RR or the hydrogen evolution reaction (HER) (Fig. S16). The variation in total current density with respect to the scan rate was also investigated in CO_2_-saturated electrolyte (Fig. S12). A clear increase in current density was observed with increasing scan rates, indicating a diffusion-controlled electrochemical process. Notably, the current response began to exhibit a sharper rise at scan rates exceeding 60 mV s^−1^, suggesting enhanced kinetics or increased accessibility of active sites at higher scan rates. This behavior further supports the electrocatalytic nature of the system under CO_2_ and may imply a transition from kinetic to mass transport limitations at elevated scan speeds.

A substantial increase in current density was observed upon CO_2_ saturation, along with the appearance of additional reduction waves in the LSV at approximately –0.93 V and –1.53 V *vs.* NHE. These features emerged prominently at scan rates exceeding 60 mV s^−1^, replacing the broad, less defined wave centered around −1.44 V *vs.* RHE observed under inert conditions. The emergence of sharper, well-defined waves under these conditions suggests that the immobilized catalyst on carbon paper actively facilitates the electrochemical reduction of CO_2_, likely through multiple electron-transfer steps. The scan rate dependence also points to a dynamic interplay between kinetic and mass transport processes, with higher scan rates enhancing access to catalytic sites and possibly promoting the formation of specific CO_2_ reduction intermediates. These findings confirm the electrocatalytic activity of the system and provide insight into its potential mechanism under operational conditions.

Electrochemical impedance spectroscopy (EIS) was conducted capturing the impedance spectrum within the frequency range of 10^6^ Hz to 0.01 Hz, with a perturbation amplitude of 10 mV (Fig. S23). This method was employed to investigate (Cl-B-SubPc) 1 and (Cl-B-SubPc-OC_12_H_23_) 2 for carbon dioxide reduction electrolysis. Initially, two platinum electrodes were employed in a single-cell configuration with the corresponding electrolyte, serving as a control experiment to ascertain the electrolyte resistance. Subsequently, the setup was transitioned to an H-cell configuration with a Nafion membrane, enabling the determination and subtraction of the membrane resistance from the electrolyte resistance.

**Fig. 4:**
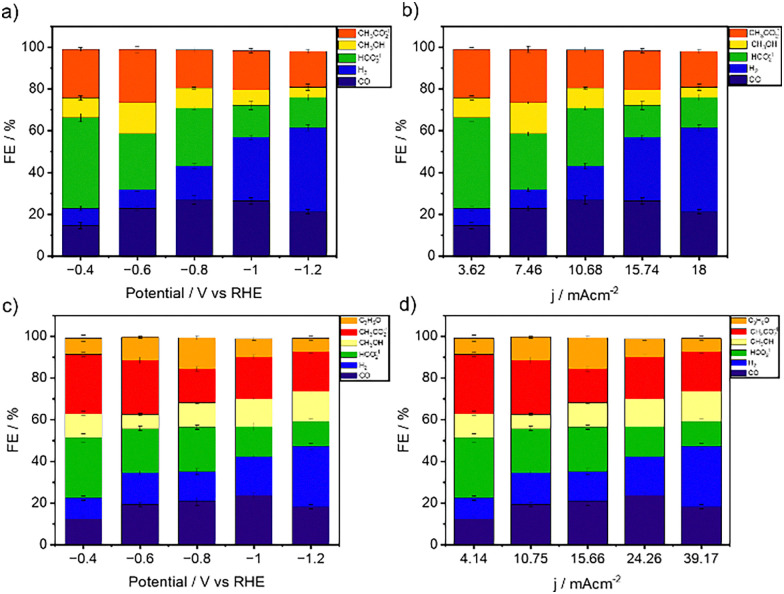
H-cell electrolysis experiments. (a) Faradaic efficiencies for CO, H_2_, HCOO^−^, CH_3_OH, and CH_3_COO^−^ obtained during one-hour electrolysis of (Cl-B-SubPc) 1 at room temperature on a modified carbon paper electrode in 0.1 M CO_2_-saturated CsHCO_3_ solution. (b) Faradaic efficiencies of (Cl-B-SubPc) 1 for CO, H_2_, HCOO^−^, CH_3_OH, and CH_3_COO^−^, obtained during one-hour electrolysis at each current density at room temperature on a modified carbon paper electrode in 0.1 M CO_2_-saturated CsHCO_3_ solution. (c) Faradaic efficiencies for CO, H_2_, HCOO^−^, CH_3_OH, CH_3_COO^−^, and C_3_H_6_O obtained during one-hour electrolysis of (Cl-B-SubPc-OC_12_H_23_) 2 at room temperature on a modified carbon paper electrode in 0.1 M CO_2_-saturated CsHCO_3_ solution. (d) Faradaic efficiencies of (Cl-B-SubPc-OC_12_H_23_) 2 for CO, H_2_, HCOO^−^, CH_3_OH, CH_3_COO^−^, and C_3_H_6_O obtained during one-hour electrolysis at each current density at room temperature on a modified carbon paper electrode in 0.1 M CO_2_-saturated CsHCO_3_ solution.

Further experiments involved replacing one platinum electrode with a carbon paper electrode as the working electrode. Lastly, the carbon paper coated with (Cl-B-SubPc) 1 and (Cl-B-SubPc-OC_12_H_23_) 2 served as the working electrode for the complete electrochemical cell evaluation through EIS (Fig. S23). The resulting fitted and calculated impedance data are summarized in Table S1. A Bode plot illustrating the behavior of the two-electrode system is presented in Fig. S23. Additionally, resistance values for each cell component (electrolyte solution, membrane, carrier electrode) in carbon dioxide reduction cell systems are summarized in Table S1. The detailed characterization based on EIS revealed negligible losses in the applied electrochemical cells. Controlled potential electrolysis (CPE) was then performed at various potentials, ranging from −0.4 to −1.2 V *vs.* RHE, over 24 hours, resulting in current densities ranging from 3.62 to 18.0 mA cm^−2^ (see Fig. 4). Electrocatalysis experiments were first conducted in a two-compartment H-cell (Fig. S22) to establish a benchmark under well-defined laboratory conditions, where both gaseous and liquid products could be quantified reliably. This configuration, while limited by ohmic resistance and mass transport, provides a widely accepted platform for initial catalyst screening and facilitates direct comparison with previously reported systems. Product analysis, conducted *via*^1^H-NMR spectroscopy and gas chromatography (GC-BID), revealed formate, methanol, and acetate as the predominant liquid products, with CO and H_2_ identified as the gaseous compounds (see Fig. S24). Two-compartment H-cell electrolysis tests of (Cl-B-SubPc) 1 on carbon paper electrodes exhibited the illustrated faradaic efficiencies of CO, H_2_, HCOO^−^, CH_3_OH, and CH_3_COO^−^ at different reductive potentials (Fig. 4a and b). Remarkably, the (Cl-B-SubPc) 1 complex on the carbonaceous electrode shows high FE_HCOO–_ = 43.45%, FE_CH_3_CO_2__^−1^ = 23.19%, and FE_Methanol_ = 9.4% at a low reductive potential of −0.4 V *vs.* RHE.

The FE_CO_ increased from −0.4 to −0.8 V *vs*. RHE and reached the maximal value (26.92%) at – 0.8 V *vs.* RHE for (Cl-B-SubPc) 1/CB. (Cl-B-SubPc) 1/CB exhibited excellent selectivity for electrochemical reduction of CO_2_ to CO, CH_3_OH, and CH_3_CO_2_^−1^ with suppressed HER, as well (Fig. 4a), resulting in high FEs for HCO_2_^−1^.

Furthermore, both the FE and current density remained relatively constant over 24 hours of CPE (Fig. 4d). CO exhibited faradaic efficiencies ranging from 14.56 to 26.92%, with product formation decreasing independently towards more negative potentials (see Fig. 4a).

At −0.4 V *vs.* RHE, formate production was favored with a substantial increase in its FE (43.45%; Fig. 4a). The faradaic efficiency of methanol production was 14.8.4% at *E*_cat_ = −0.6 V *vs.* RHE, which decreased to 9.5% at *E*_cat_ = −0.8 V *vs.* RHE (see Fig. 2c). The most elevated efficiency in CH_3_OH, and CH_3_CO_2_^−1^ production was achieved with a selectivity of 14.8 and 25.5% at −0.6 V *vs.* RHE, while CO formation exhibited a selectivity of 26.9% at −0.8 and −1.0 V *vs.* RHE.

The faradaic efficiencies for (Cl-B-SubPc-OC_12_H_23_) 2/CB are presented in Fig. 4c. The major products were carbon monoxide (CO), hydrogen (H_2_), formate (HCOO^−^), methanol (CH_3_OH), acetate (CH_3_COO^−^), and acetone (C_3_H_6_O). The faradaic efficiency of methanol production delivers 11.67% at a potential of −0.4 V *vs.* RHE, decreases down to 6.73% as the potential negatively goes to −0.6 V *vs.* RHE, and then gradually ramps up to a maximal value of 14.5% with further negatively increasing potential to −1.2 V *vs.* RHE (Fig. 4c). With the increase of applied potential, the overall faradaic efficiency of CO increases first and then decreases.

Evidently, (Cl-B-SubPc-OC_12_H_23_) 2 exhibited the highest selectivity for liquid products among all samples, but a slight decrease in the efficiency of CO formation in comparison with (Cl-B-SubPc) 1.

The stability assessment of (Cl-B-SubPc) 1/CB was conducted through extended chronoamperometry at −1.0 V. As depicted in Fig. S 27, neither the current density nor the faradaic efficiency (FE) of the liquid product exhibited a noticeable decline over a duration of six days of electrolysis, showcasing the stability of (Cl-B-SubPc) 1/CB. In a final observation, it is however suggested that the electrocatalyst–electrolyzer architecture could be even further optimized regarding its stability as the system's FE experienced a minor drop from 75.43% at −1.0 *vs.* RHE to 71.3% after 180 hours of reaction.

To verify CO is reduced from CO_2_, the control electrocatalysis was carried out in an Ar-saturated electrolyte. Control experiments in an Ar-saturated or N_2_-saturated electrolyte were widely used to verify that the carbon source of the reduced product comes from CO_2_ electroreduction in many studies.^45,46^ If no hydrocarbon product was detected in an Ar-saturated or N_2_-saturated electrolyte, proving that the source of the hydrocarbon product is CO_2_ gas. It was found that no products were detected (Fig. S35), confirming the resulting products were produced from CO_2_.^47^

The catalytic cycle is initiated by the electrochemical reduction of compounds 1 and 2 at applied potentials ranging from –0.8 V to –1.0 V *versus* the normal hydrogen electrode (NHE). This reduction is irreversible and results in the dissociation of the axially coordinated chloride ligand, generating a reactive anion radical species, [B-SubPc]˙^−^. As noted above, the X-ray photoelectron spectrum in Fig. 2g provides direct evidence for the loss of the axial chloride ligand, thereby confirming dissociation upon reduction. Operando UV-vis spectroelectrochemical analysis reveals significant spectral changes, including broadening and flattening of absorption bands in the 400–600 nm region, as well as the appearance of a new, broad absorption feature centered around 650–700 nm (Fig. 4a and b). These observations suggest extensive electronic reorganization, which likely facilitates subsequent catalytic transformations.

Chemical reduction of 1 using potassium graphite (KC_8_) in tetrahydrofuran (THF) similarly results in the loss of the axial chloride ligand. Comparative ^11^B NMR spectroscopy of Cl–B–SubPc (1) before and after chemical reduction shows a shift from –13.48 ppm to a downfield resonance at –2.78 ppm (Fig. S28).

Product identification and quantification were conducted using gas chromatography (GC) for gaseous products and ^1^H- and ^13^C-NMR spectroscopy for liquid products. As shown in Fig. 4a and b, electrocatalytic CO_2_ reduction with catalyst 1 over the potential range of −0.4 V to −1.2 V *vs.* RHE produces a mixture of formate, acetate, methanol, carbon monoxide, and hydrogen. At more negative potentials (≥−1.0 V *vs.* RHE), CO and H_2_ evolution dominates, indicating a shift in selectivity toward gas-phase products. This suggests that at higher overpotentials competitive proton reduction and CO_2_-to-CO conversion become the predominant pathways.

For (Cl-B-SubPc-OC_12_H_23_) 2, acetone is additionally detected at −0.8 V to −1.0 V *vs.* RHE, suggesting a distinct mechanistic pathway akin to those proposed by Koper *et al.* and Chen *et al.*^23^ These differences in product selectivity emphasize the crucial role of catalyst structure and electronic properties in steering CO_2_ reduction pathways. The following section explores the mechanistic details underlying these observations.

**Fig. 5:**
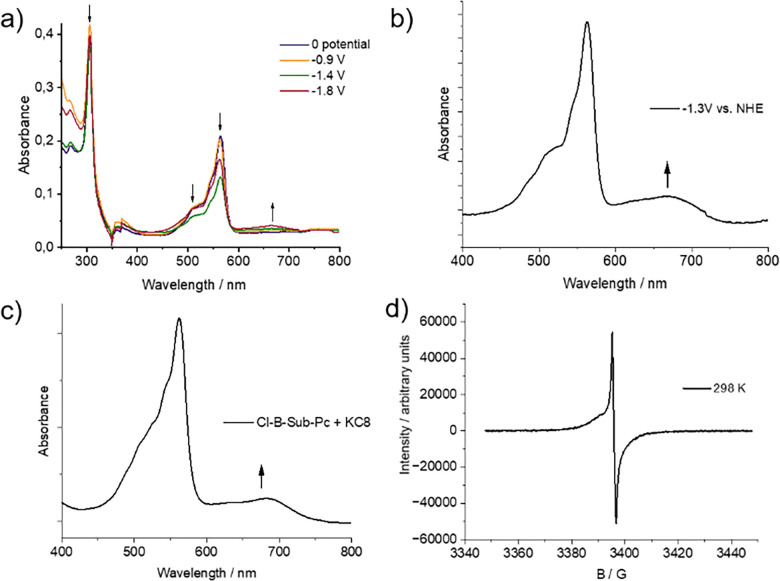
(a) Spectroelectrochemistry (UV-vis EC) of (Cl-B-SubPc) 1 in DCM at different reductive potentials, (b) UV-vis EC at −1.3 V *vs.* NHE, (c) chemical reduction of (Cl-B-SubPc) 1 with KC_8_ in THF, and (d) ESR spectrum of (Cl-B-SubPc) 1 after reduction with KC_8_ in THF at 298 K.

**Fig. 6:**
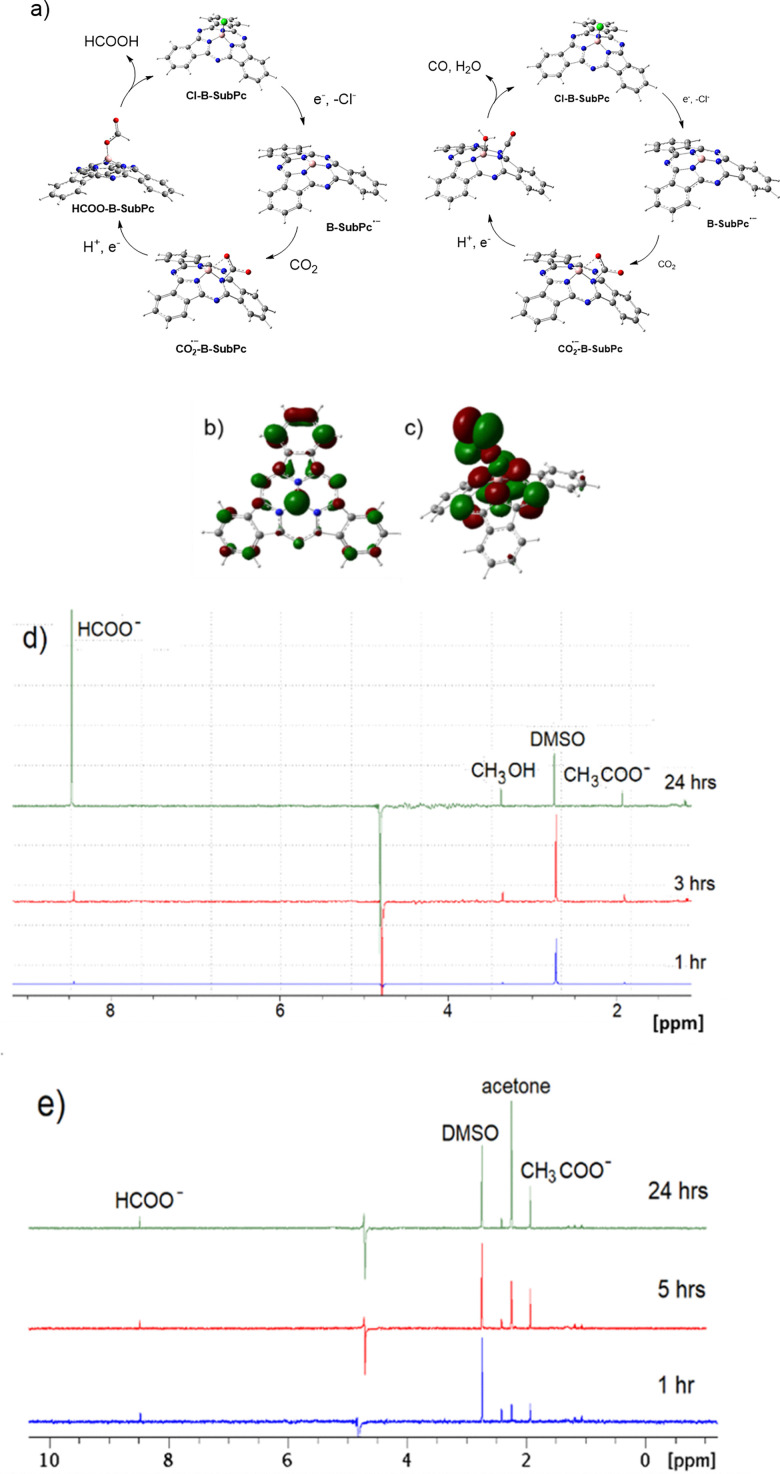
(a) Schematic illustration of the proposed mechanisms for electrocatalytic CO_2_ reduction to formate and CO. (b) Frontier molecular orbital representation of [B-SubPc]˙^−^, highlighting the unoccupied orbital at the boron center, which contributes to its Lewis acidic and oxophilic character within the boron subphthalocyanine complex. (c) Electron density map showing the bonding molecular orbital of the H-COO-B-SubPc intermediate. (d) ^1^H NMR spectra showing the formation of formate (*δ* = 8.44 ppm), methanol (*δ* = 3.33 ppm), and acetate (*δ* = 1.93 ppm) after 24 hours of electrocatalysis with (Cl-B-SubPc) 1 at −0.8 V *vs.* RHE. (e) ^1^H NMR spectra revealing the formation of formate (*δ* = 8.44 ppm), acetone (*δ* = 2.24 ppm), and acetate (*δ* = 1.93 ppm) under the same electrocatalytic conditions with (Cl-B-SubPc-OC_12_H_23_) 2. DMSO is used as an internal standard (*δ* = 2.74 ppm).

The mechanistic pathway towards formate production is illustrated in Fig. 6a. Step 1: catalyst activation *via* electron transfer. At the cathode, the Cl-B-SubPc catalyst undergoes an initial one-electron reduction, forming a highly reactive anion radical: B-SubPc + e^−^ → [B-SubPc]˙^−^. Electron spin resonance (ESR) spectroscopy provides direct evidence for the formation of this anion radical species, characterized by a distinctive dispersive signal with a *g*-value of 2.003, consistent with a free electron (Fig. 5d). This signal confirms extensive delocalization of the unpaired electron across the SubPc π-system, a feature integral to its function as a redox-active center in CO_2_ reduction. Density functional theory (DFT) calculations further reveal an increased electron density at the macrocycle, facilitating its interaction with CO_2_ (Fig. S31). The boron center is highly Lewis acidic and oxophilic (Fig. 6b). Step 2: CO_2_ activation *via* nucleophilic attack. The reduced [B-SubPc]˙^−^ species interacts with CO_2_ through nucleophilic attack, leading to the formation of a bent CO_2_ adduct: [B-SubPc]˙^−^+ CO_2_ → CO_2_˙^−^−B-SubPc. This interaction is critical in lowering the energy barrier for subsequent protonation and stabilizing the CO_2_ reduction intermediate.

Step 3: first protonation. A proton donor, such as water or a weak acid, delivers a proton to the activated CO_2_ complex, yielding the HCOO-B-SubPc intermediate: [CO_2_˙^−^−B-SubPc] + H^+^ → HCOO-B-SubPc. Step 4: second electron transfer and formate release. A second electron transfer enables the cleavage of the HCOO-B-SubPc bond, releasing the formate product and regenerating the catalyst: HCOO-B-SubPc + e− → HCOO− + B-SubPc. This step ensures catalyst recyclability and enables continuous electrocatalytic operation. The carbon monoxide formation proceeds through the initial one-electron reduction of the Cl-B-SubPc catalyst, again forming the highly reactive anion radical: B-SubPc + e^−^ → [B-SubPc]˙^−^. The reduced [B-SubPc]˙^−^ species interacts with CO_2_ through nucleophilic attack, leading to the formation of a bent CO_2_ adduct: [B-SubPc]˙^−^ + CO_2_ → CO_2_˙^−^−B-SubPc. Subsequent proton-coupled electron transfer (PCET) leads to the CO-H_2_O-B-subPC intermediate, which splits off the CO and H_2_O molecules from the B-SubPC complex (Fig. S31). The mechanistic details of methanol, acetate, and acetone production are discussed in the SI.

**Fig. 7:**
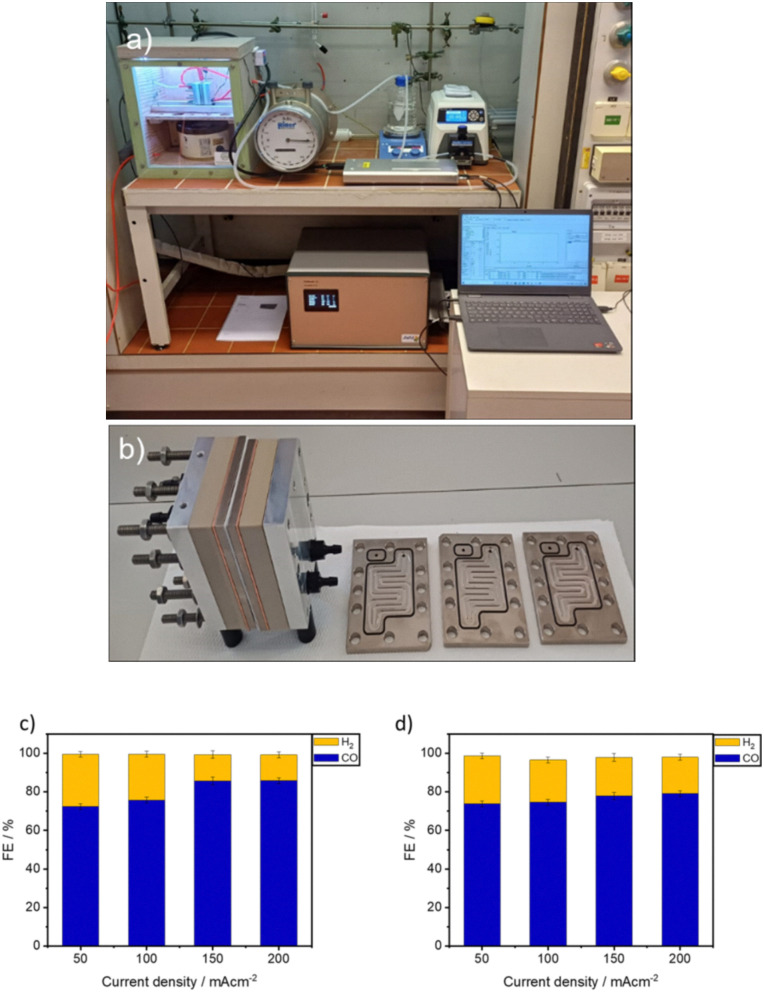
(a) Image of our custom zero-gap electrolyzer setup used in our work. (b) Zero-gap electrolyzer cell stack. (c) Performance of (Cl-B-SubPc) 1 for CO_2_ reduction at 50, 100, 150, and 200 mA cm^−2^ at 60 °C after two hours of electrolysis. All investigated GDEs contained a catalytic loading of 1 mg cm^−2^ of the active material. (d) Performance of (Cl-B-SubPc-OC_12_H_23_) 2 for CO_2_ reduction at 50, 100, 150, and 200 mA cm^−2^ at 60 °C after two hours of electrolysis. All investigated GDEs contained a catalytic loading of 1 mg cm^−2^ of the active material.

The long-term stability of the radical anion is critical for maintaining high catalytic efficiency, as a prolonged electrochemical operation may lead to catalyst deactivation. The combined spectroelectrochemical and ESR data strongly support this mechanistic framework, highlighting (Cl-B-SubPc) 1 and (Cl-B-SubPc-OC_12_H_23_) 2 as a highly effective molecular electrocatalyst for CO_2_ conversion. Further investigations are warranted to explore the interplay between ligand modifications, electronic structure tuning, and catalytic performance to refine its application in sustainable carbon capture and utilization strategies. In order to further investigate the practical applicability of the investigated catalytic system, final measurements were conducted in a zero-gap cell electrolyzer. To complement these studies, the catalysts were subsequently evaluated in a zero-gap electrolyzer, which more closely reflects the practical device operation. In contrast to the H-cell, the zero-gap configuration minimizes ion transport limitations, sustains higher current densities, and alters the local reaction environment at the electrode–electrolyte interface. As a result, the product selectivity observed in the zero-gap device differs significantly from that obtained in the H-cell, an effect that is consistent with prior reports on both molecular and heterogeneous electrocatalysts.^22^ The home-built zero-gap cell electrolyzer employed here consisted of flow plates, sample and IrO_2_ electrodes, Teflon spacers, and a PiperION anion exchange membrane (40 microns) (Fig. 7a and b). In pursuit of cost reduction for overall CO_2_ capture and conversion systems, attention is directed not only towards optimizing CO_2_ electrochemical reactors but also towards the capture and release of CO_2_ to the electrochemical cell. The previously considered inefficient KOH reduction, whose applicability was in doubt, is now gaining attention as one of the most promising routes to developing an efficient integrated CO_2_ capture and conversion system involving the electrochemical reduction of CO_2_.^23^

Despite the promising findings in terms of cathodic and full cell electrical efficiency, the stability of the catalyst is crucial for commercial applications. Commercial gas diffusion electrodes (GDEs) are known to suffer from stability issues, losing hydrophobicity and experiencing flooding over time. This behavior is exacerbated under pressure.^25^

All zero-gap cell experiments related to CO_2_ electroreduction were conducted using an electrochemical configuration as illustrated in Fig. 7b. The cathode gas diffusion electrode (GDE), coated with the catalyst (geometric active area of 9 cm^2^ with a catalyst loading of 1 mg cm^−2^), was separated from the anode by an anion exchange membrane (PiperION A40-HCO_3_). The membrane was conditioned overnight in 1 M KOH and washed with Milli-Q water before electrolysis. The employed anode featured a loading of 1 mg cm^−2^ IrO_2_. 0.1 M CsOH was employed as the anolyte and circulated through the anode flow channels, while gaseous CO_2_ was fed into the cell on the cathode side. Utilizing a temperature-controlled humidifier, the relative humidity of the CO_2_ gas was adjusted based on the applied current density. For each CO_2_ reduction experiment, fresh electrolyte was prepared and circulated through the electrochemical cell using peristaltic pumps at a rate of 50 mL min^−1^. An automatic mass flow controller maintained the flow of the input CO_2_ (99.99%) at 100 sccm throughout each experiment.

Regarding the results obtained in the zero-gap cell, the identified optimal conditions – a CD of 200 mA cm^−2^, high flow rate (50 mL min^−1^), 60 °C operation, and low CsOH concentration (0.1 M) – demonstrated a maximum FE of 85.72% for (Cl-B-SubPc) 1 and 79.08% for (Cl-B-SubPc-OC_12_H_23_) 2. These findings offer promising insights for developing finely designed CsOH electrolyzers for the integrated capture and conversion of CO_2_. Additionally, the stability of 1 was examined at 100 mA cm^−2^ over the course of 24 hours, which revealed a stable cell voltage of *ca.* 3.6 V in addition to just a minor drop in FE for CO of 0.14% h^−1^ from 75.7 to 72.4% (compare Fig. S29 and S30).

## Conclusions

This work introduces molecular boron subphthalocyanine chlorides, Cl–B–SubPc 1 and Cl–B–SubPc–OC_12_H_23_2, as efficient molecular electrocatalysts for the selective electrochemical reduction of CO_2_ to C_1_–C_3_ products. Employing both H-cell and zero-gap electrolyzer configurations, the systems enable the formation of liquid-phase products (formate, methanol, acetate, and acetone) and gas-phase products (CO and H_2_), as verified through comprehensive spectroscopic and electrochemical characterization. Mechanistic investigations reveal that an irreversible one-electron reduction promotes the dissociation of the axial chloride ligand, yielding a catalytically competent anion radical species. This intermediate facilitates CO_2_ activation *via* proton-coupled electron transfer (PCET) pathways. Operando UV-vis spectroelectrochemistry and electron spin resonance (ESR) spectroscopy provide compelling evidence for the generation and persistence of this reactive species under electrochemical conditions.

Crucially, performance evaluation in a zero-gap electrolyzer demonstrates high catalytic activity and selectivity: catalyst 1 achieves a faradaic efficiency of 85.72% for CO at a current density of 200 mA cm^−2^, while catalyst 2 reaches 79.08% under comparable conditions. These results underscore the industrial relevance of these molecular systems for efficient CO production. Collectively, this study establishes a promising new class of boron-based molecular electrocatalysts for CO_2_ reduction and provides a mechanistic foundation for the development of scalable strategies in carbon valorization and renewable energy storage.

## Author contributions

Conceptualization: WS. Formal analysis: FY, SO, SV, MS, and DK. Funding acquisition: WS. Investigation: FY, SO, and SV. Methodology: FY, DK, and WS. Project administration: WS. Resources: WS. Supervision: WS. Validation: FY, DK, and WS. Visualization: FY and WS. Writing – original draft: FY and WS. Writing – review & editing: FY, DK, and WS.

## Conflicts of interest

There are no conflicts to declare.

## Supplementary Material

YA-004-D5YA00136F-s001

## Data Availability

The datasets supporting this article have been included as part of the SI. See DOI: https://doi.org/10.1039/d5ya00136f. Further information that is not provided can be obtained from the authors upon request.
