# Supplementary material for: Boron subphthalocyanine complexes for CO2 electroreduction: molecular design and catalytic insights
Source: Energy Adv. 2025 Sep 5;4(10):1241–50. doi: 10.1039/d5ya00136f (PMC12455475; doi:10.1039/d5ya00136f)
Supplement: YA-004-D5YA00136F-s001 [file YA-004-D5YA00136F-s001.pdf]

Supplementary Information (SI) for Energy Advances.
This journal is © The Royal Society of Chemistry 2025

Supplementary Informations

Boron Subphthalocyanine Complexes for CO_2 Electroreduction: Molecular Design and Catalytic Insights

Farzaneh Yari,[a] Simon Offenthaler,[a] Sankit Vala, [a] Dominik Krisch[a], Markus Scharber[b] and Wolfgang Schöfberger*[a]

[a] Farzaneh Yari, Simon Offenthaler, Sankit Vala, Dominik Krisch, Prof. Dr. W. Schöfberger
Institute of Organic Chemistry, Laboratory for Sustainable Chemistry and Catalysis (LSusCat)
Johannes Kepler University (JKU)
Altenberger Straße 69, 4040 Linz, Austria.
E-mail: wolfgang.schoefberger@jku.at
Homepage: https://www.jku.at/en/institute-of-organic-chemistry/team/schoefberger-lab

[b] Prof. Dr. M. C. Scharber Institute of Physical Chemistry and Linz Institute of Organic Solar Cells (LIOS)
Johannes Kepler University Linz
Altenberger Straße 69, 4040 Linz, Austria

Supporting information for this article is given via a link at the end of the document.

Keywords: CO_2 electrocatalysis • boron subphthalocyanine • H-cell electrolyzer • zero-gap cell electrolyzer

Content

1. Synthesis of (Cl-B-SubPc) 1 2
2. Synthesis of (Cl-B-SubPc-$OC_{12}H_{23}$) 2 3
3. Structural characterization of the catalysts (XPS, NMR, UV-vis) 5
4. Electrochemical characterization 11
5. Characterization by Electrochemical Impedance Spectroscopy (EIS)- Method 17
6. Product analysis by GC-BID 18
7. Product detection and quantification by ^{1}H-NMR 20
8. Spectroelectrochemistry and chemical reduction experiments 26
9. Geometry Optimization, Mechanistic Insights 27
10. References 31

1. Synthesis of (Cl-B-SubPc) 1

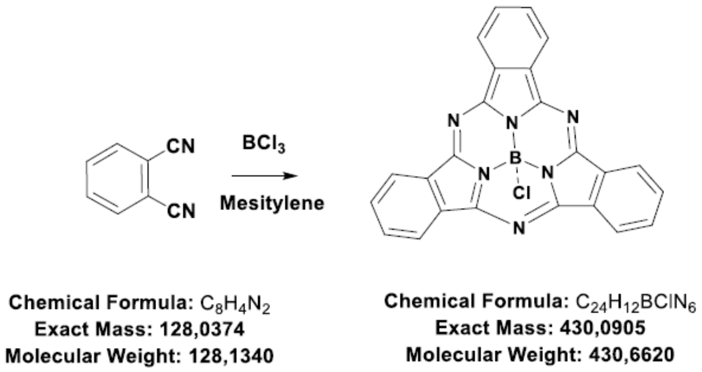

Figure S1. Chemical synthesis of the (Cl-B-SubPc) **1**.

Phthalonitrile (0.500 g, 3.90 mmol) was dissolved in mesitylene (20.0 mL) under an argon atmosphere. To this solution, boron trichloride (7.50 mL, 1.0 M in CH_2Cl_2) was added dropwise with stirring. The resulting mixture was heated to reflux and stirred for 2 h. Reaction progress was monitored by thin-layer chromatography (TLC) on silica gel using *n*-heptane/dichloromethane (1:3) as the eluent. After completion, the reaction mixture was allowed to cool to room temperature, and the solvent was removed under reduced pressure using a rotary evaporator. The resulting crude solid was dried overnight in a desiccator. The dried crude product was subsequently redissolved in toluene (200 mL) and concentrated again by rotary evaporation. The solvent was removed under vacuum to yield Cl-SubPc **1** as a pink solid (0.4 g, 73 %).[1]

^{1}H NMR (500 MHz, THF-d_8): δ_{1H} (ppm) = 8.86-8.91 (6H, m), 7.91-7.95 (6H, m). ^{11}B NMR (160.4 MHz, THF-d_8): δ_{11B}(ppm) = -13.6. ^{13}C NMR (125.7 MHz, THF-d_8, 25°C) δ_C(ppm) = 149.7 (-C=N: inner core carbons), 125.7 (-C=C: iminoisoindoline unit), 122 (nonperipheral-C), 119.8 (peripheral C).

HRMS [M+H$^+$] exact mass calculated for $C_{24}H_{12}BN_6Cl$ + H: m/z 431.0975 found 431.0972.

2. Synthesis of (Cl-B-SubPc-$OC_{12}H_{23}$) 2

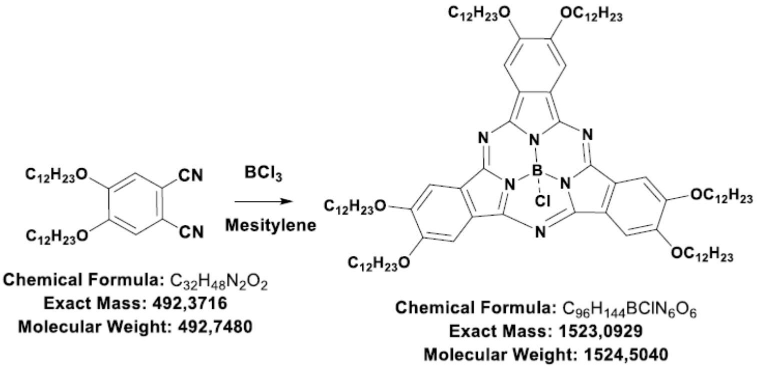

Figure S2. Chemical synthesis of (Cl-B-SubPc-$OC_{12}H_{23}$) **2**.

2,5-bis(dodecyl)phthalonitrile (0.74 g, 1.6 mmol) was dissolved in mesitylene (30.0 mL) under an argon atmosphere. To this solution, boron trichloride (9.50 mL, 1.0 M in CH_2Cl_2) was added dropwise with stirring. The resulting mixture was heated to reflux and stirred for 2 h. Reaction progress was monitored by thin-layer chromatography (TLC) on silica gel using *n*-heptane/dichloromethane (1:3) as the eluent. After completion, the reaction mixture was allowed to cool to room temperature, and the solvent was removed under reduced pressure using a rotary evaporator. The resulting crude solid was dried overnight in a desiccator. The dried crude product was subsequently redissolved in toluene (200 mL) and concentrated again by rotary evaporation, and the resultant purple solid was purified with silica gel column chromatography using heptane: ethyl acetate (3:1, v:v, Rf: 0.68).[1] The solvent was removed under vacuum to yield Cl-SubPc **2** as a pink solid. (0.7 g, 85 %)

^{1}H NMR (500 MHz, $CDCl_3$): δ_H (ppm) = 7.7 (s, 6H), 4.0 (t, 12H), 1.8-1.3 (m, 108H), 0.9 (18H). HRMS [M+H$^+$] exact mass calculated for $C_{96}H_{144}BClN_6O_6$ +H: m/z 1523.0929 found 1523.0962.

Experimental Details

The catalytic loading was determined by weighing before and after spray-coating as 0.2 mg cm^{-2}. Note that for XPS characterization, the GDLs were prepared similarly only without the addition of Nafion in order to avoid suppression of other elements' intensity by mainly carbon and oxygen. The electrochemical properties of the electrocatalysts were systematically examined through both homogeneous and heterogeneous approaches. Homogeneous electro-characterization was specifically employed to assess catalyst responsiveness to CO_2 and involved cyclic voltammetry measurements. Glassy carbon served as the working electrode, a Pt wire as counter and a AgCl coated Ag wire as pseudo reference electrode in 10 mL of DCM with 0.1 M TBAP as the supporting electrolyte, employing a scan rate of 30 mV s^{-1}. Conversely, heterogeneous measurements were conducted to elucidate electrochemical phenomena that could be encountered in scaled-up applications. All heterogeneous electrochemical measurements except for zero-gap cell experiments where noted, were carried out in an H-type cell, where compartments were separated by a Nafion membrane. Before measurements, the electrolyte solution (0.1 M $CsHCO_3$) was purged with CO_2 for one hour at a flow rate of 50 mL min^{-1} and then bubbled continuously with CO_2 at 10 mL min^{-1} during the test until the pH of the saturated solution reached 6.8 after 1 h. 0.1 M $CsHCO_3$ was chosen as electrolyte, as H_2 formation was lowest in this case. The Boron subphthalocyanine chlorides (Cl-B-SubPc) **1** and (Cl-B-SubPc-$OC_{12}H_{23}$) **2** were physisorbed on carbon paper as a supportive electrode with an effective loading of 0.2 mg cm^{-2} and tested in a three-electrode configuration with the catalyst loaded cathodes as working, Ag/AgCl/1M KCl as reference and platinum wire as counter electrode Cyclic voltammetry (CV) measurements under heterogeneous conditions were conducted to study the electrocatalytic efficiency towards CO_2 reduction reaction (e-CO_2RR) in an aqueous electrolyte solution. They were performed under argon and CO_2 in 0.1 M $CsHCO_3$ at pH 6.8 electrolyte solution. Potentiostatic chronoamperometry (CA) was conducted to measure the consumed electrons during electrosynthesis in coulombs by integration of the current over time. Throughout the electrolysis, CO_2 gas was introduced into the cathodic compartment of the H-cell at a flow rate of 10 mL min^{-1} to maintain a CO_2-saturated environment. The voltage on the working electrode was incrementally adjusted, ranging from -0.4 to -1.2 V vs. RHE, and held steady for one hour with stirring at each potential to record the corresponding chronoamperometric curve. The electrochemical active surface area (ECSA, cm^2) was calculated by double-layer capacitance C_{DL}, which was measured by conducting CV within a 200mV window centered at -0.02 V vs. RHE for (Cl-B-SubPc) **1** and 0.1 V vs. RHE for (Cl-B-SubPc-$OC_{12}H_{23}$) **2**. All potentials were eventually transformed to the reversible hydrogen electrode reference through the following relationship:

$$E_{vs\ RHE} = E_{vs\ Ag/AgCl} + 0.209\ V + 0.0592\ V \times pH$$

The different current densities (i_c, mA cm^{-2}) were plotted as a function of scan rate (v, mV s^{-1}) with a slope equal to the C_{DL} (μF cm^{-2}). The ECSA can be obtained by comparing the correlation C_{DL} (μF) to a smooth planar surface (C_{REF}, μF cm^{-2}) which was often assumed to be 40 μF cm^{-2} following these equations: [2-4]

$$ECSA=\frac{C_{DL}}{C_{REF}}$$

$C_{DL}=C_{dl}\times S$ (S is the surface area of electrode, cm^2)

A comprehensive structural characterization was conducted using NMR, UV-Vis, and XPS techniques, verifying the catalytic structure (Figures S1-9). **1** and **2** were deposited on carbon paper through drop casting using a methanol mixture and underwent X-ray photoelectron spectroscopy (XPS) analysis both before (see Fig. S3 and S4) and after the electroreduction (refer to Fig. S6). The XPS survey scans encompassed the corresponding B1s, Cl2p, N1s, O1s binding energy regions (see Fig. S3-6). However, the XPS scans demonstrate that the catalyst remains stable throughout the course of electrocatalysis (Fig. S6).

All zero-gap cell experiments related to CO_2 electroreduction were conducted using an electrochemical configuration, as illustrated in Figure S33. The cathode gas diffusion electrode (GDE), prepared with a catalyst (geometric active area of 1 mg cm^2), was separated from the anode by an anion exchange membrane (PiperION A40-HCO3). The membrane was conditioned overnight in 1 M KOH before being washed with Milli-Q water before electrolysis. The anode employed a Ti fleece with a loading of 1 mg cm^{-2} IrO_2. A liquid electrolyte (0.1 M CsOH) was introduced into the anolyte chamber on each side of the anion exchange membrane. Gaseous CO_2 was fed into the cell behind the cathode GDE and diffused into the catalyst. Utilizing a temperature-controlled humidifier, the relative humidity of the CO_2 gas was adjusted based on the applied current density. [3,4] For each CO_2 reduction experiment, fresh electrolyte was prepared, and it was circulated through the electrochemical flow cell using peristaltic pumps at a rate of 50 mL min^{-1}. An automatic mass flow controller maintained the flow of the input CO_2 (99.99%) at 100 sccm throughout each experiment.

3. Structural characterization of the catalysts (XPS, NMR, UV-vis)

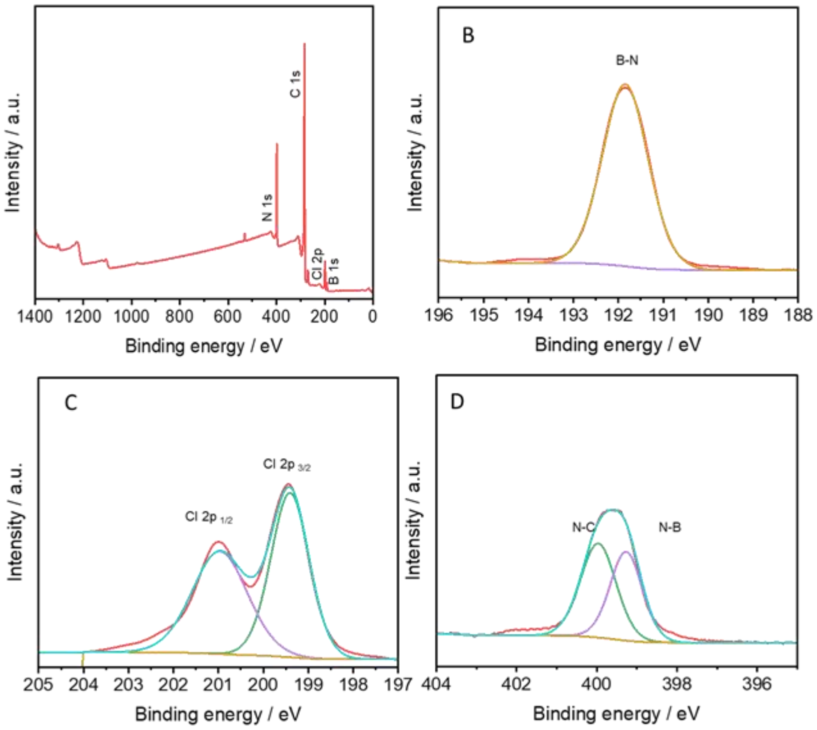

Figure S3. XPS Analysis of the (Cl-B-SubPc) **1** a) B 1s, b) N1s, and c) Cl 2p.

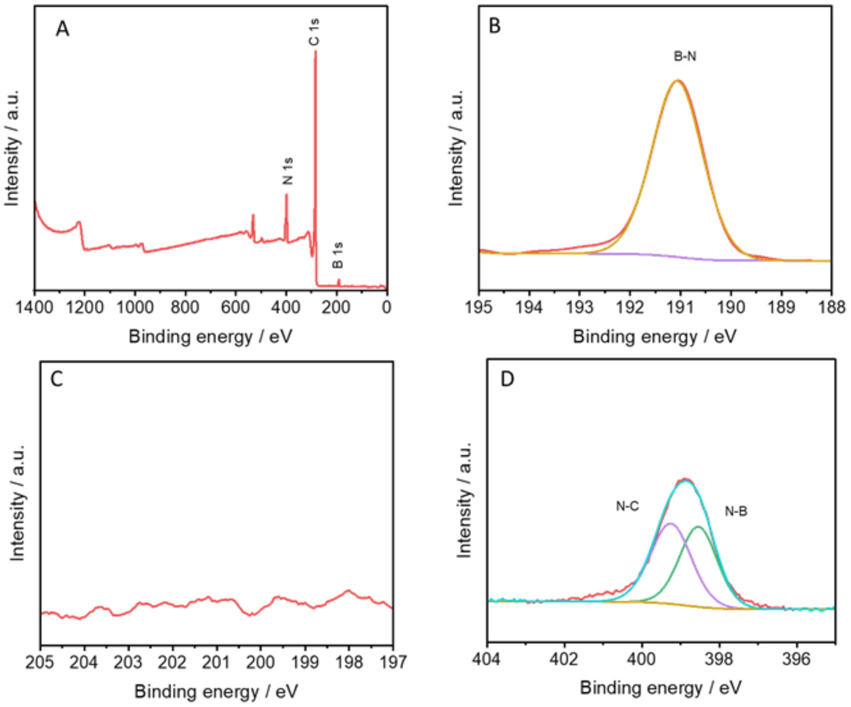

Figure S4. XPS Analysis of the (Cl-B-SubPc) **1**/CB after 24 hours of electrolysis at -1.0 vs RHE, A) B 1s, B) N 1s, C) Cl2p

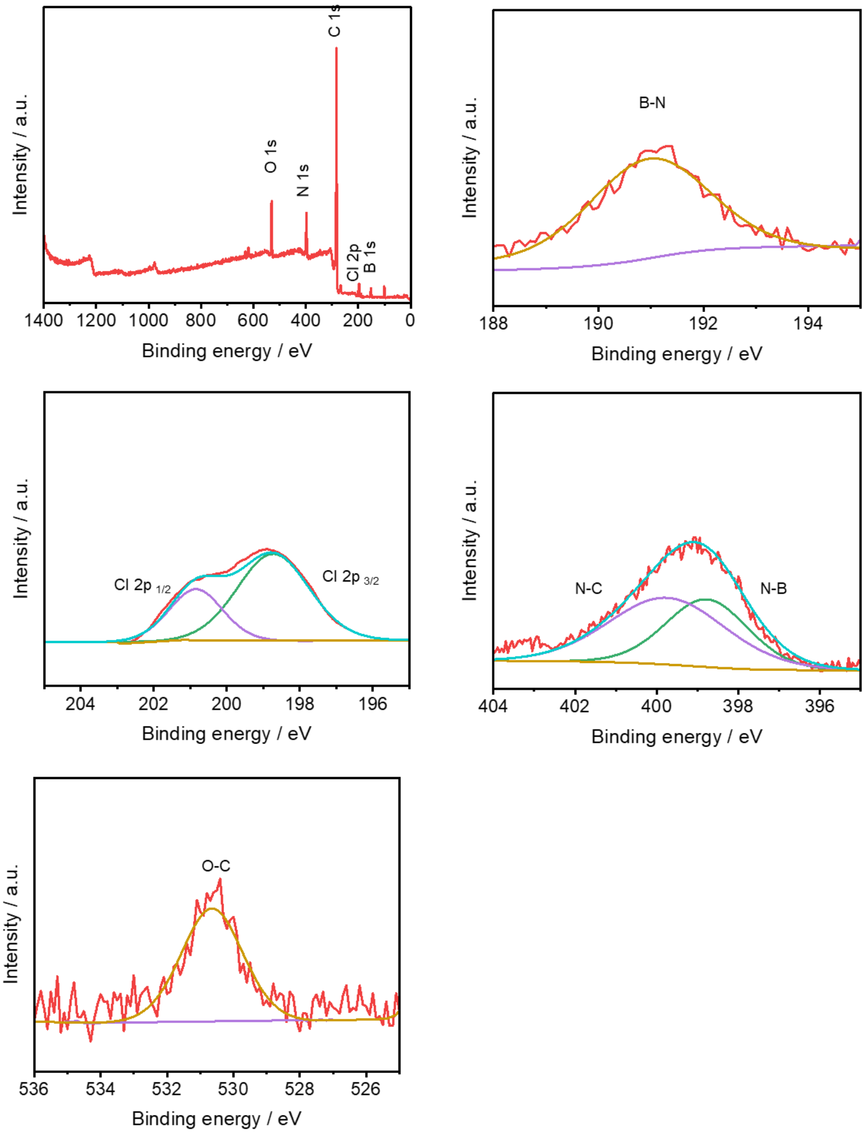

Figure S5. XPS Analysis of the (Cl-B-SubPc-$OC_{12}H_{23}$) **2** /CB, A) B 1s, B) N 1s, C) Cl2p.

.

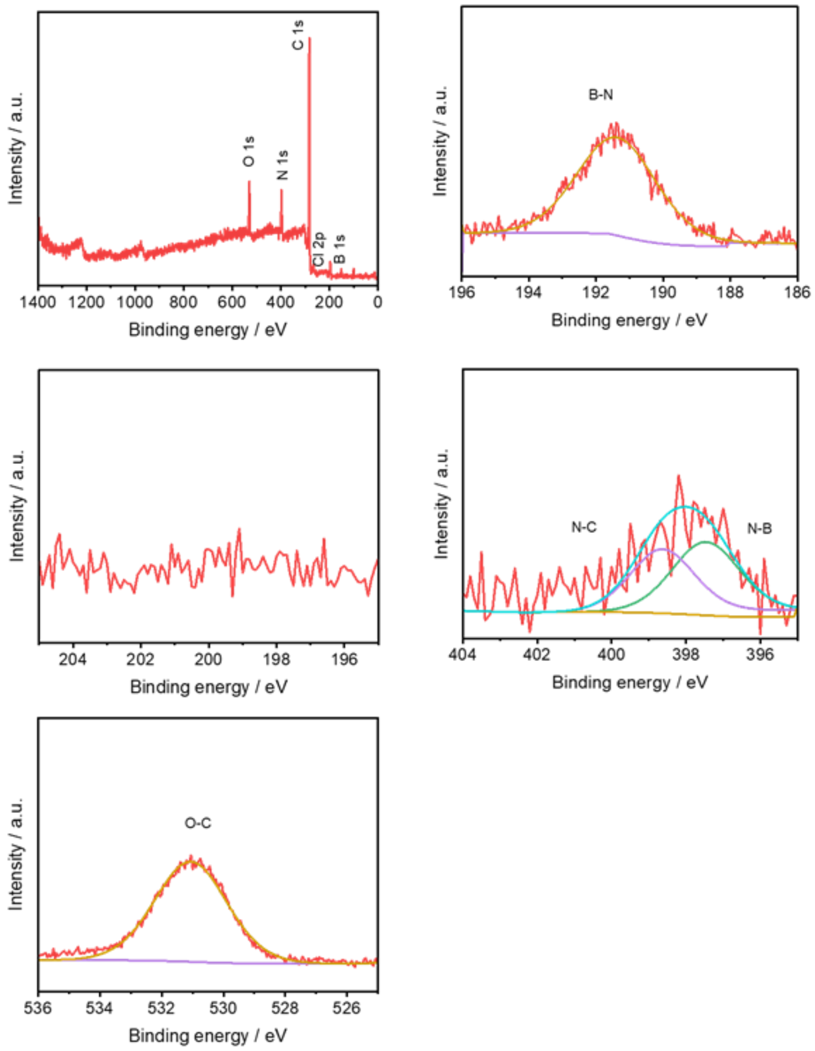

Figure S6. XPS Analysis of the (Cl-B-SubPc-$OC_{12}H_{23}$) **2** /CB after 24 hours of electrolysis at -1.0 vs RHE, A) B 1s, B) N 1s, C) Cl2p.

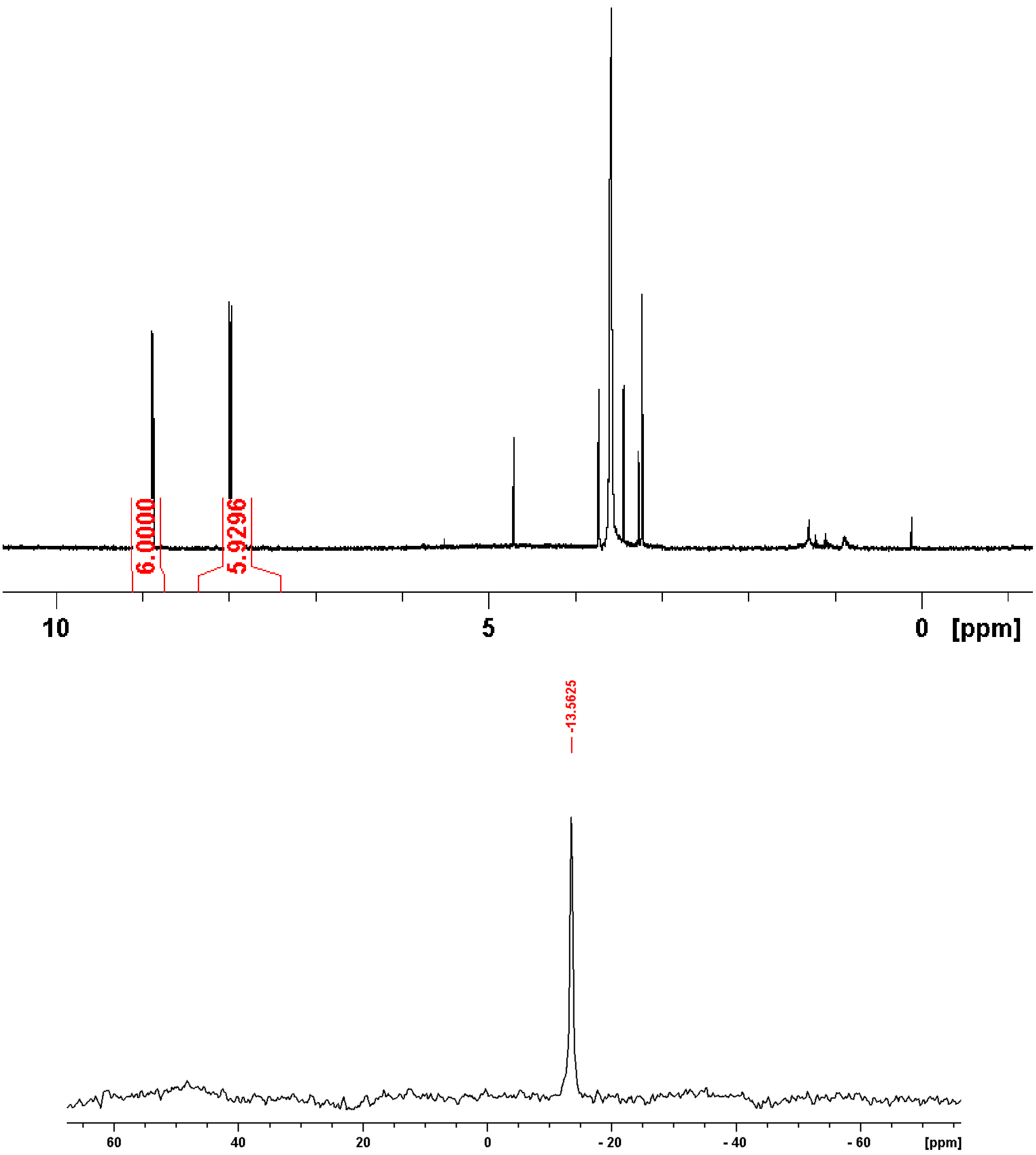

Figure S7. ^{1}H NMR and ^{11}B NMR spectra of Cl-B-SubPc **1** in THF-d_8.

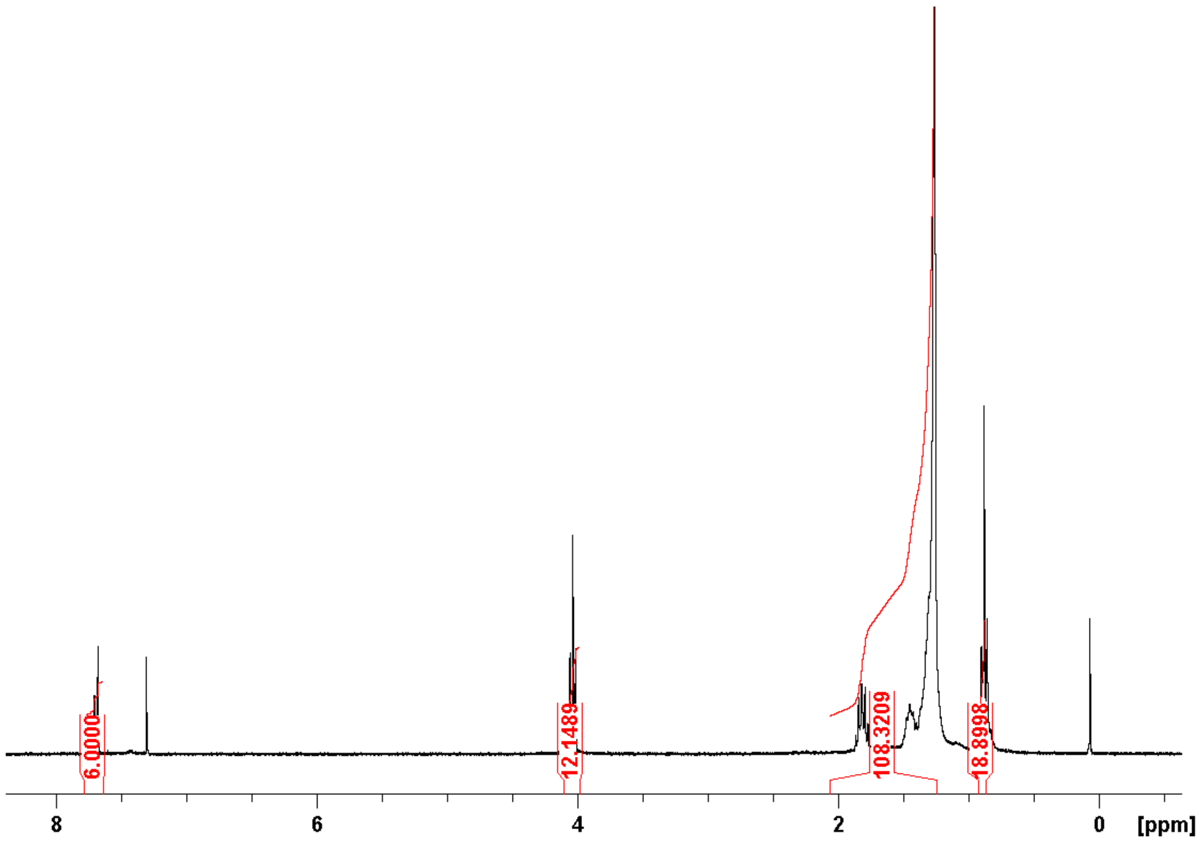

Figure S8. ^{1}H NMR spectrum of Cl-B-SubPc-$OC_{12}H_{23}$ **2** in $CDCl_3$.

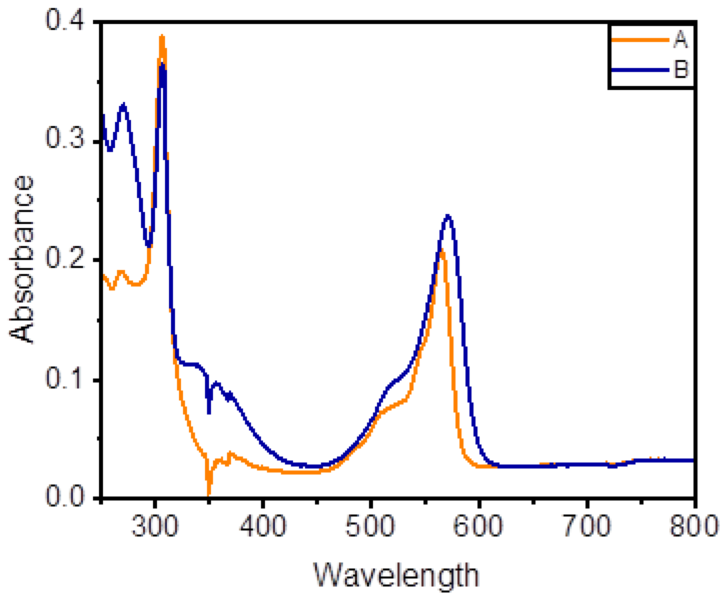

Figure S9. UV-vis of A) (Cl-B-SubPc) ***1***, λ_{max} nm: 269, 306, 562. B) (Cl-B-SubPc-$OC_{12}H_{23}$) **2**, λ_{max} nm: 271, 307, 571.

4. Electrochemical characterization

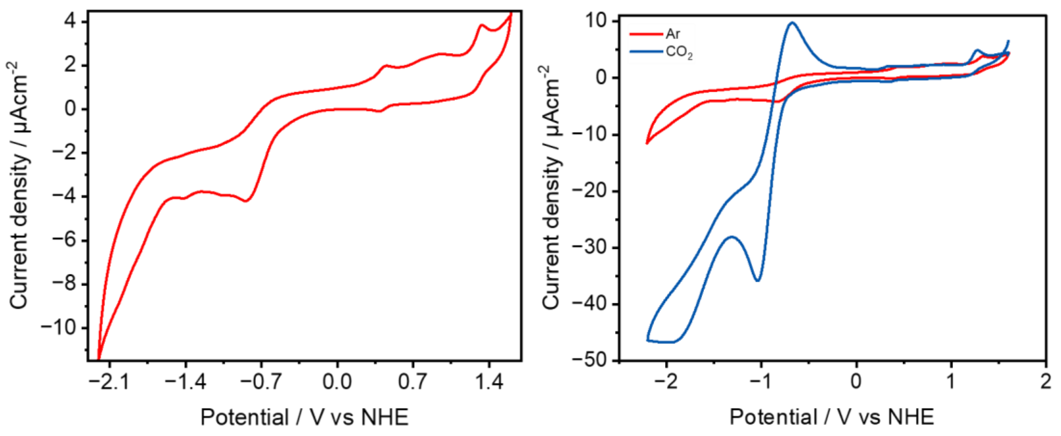

Figure S10. Comparison of cyclic voltammograms of (Cl-B-SubPc) **1** dissolved in DCM under argon and CO_2 containing 0.1 M TBAP as supporting electrolyte with glassy carbon as working, platinum wire as counter and nonaqueous pseudo-Ag/AgCl as reference electrode with a scan rate of 30 mVs^{-1}.

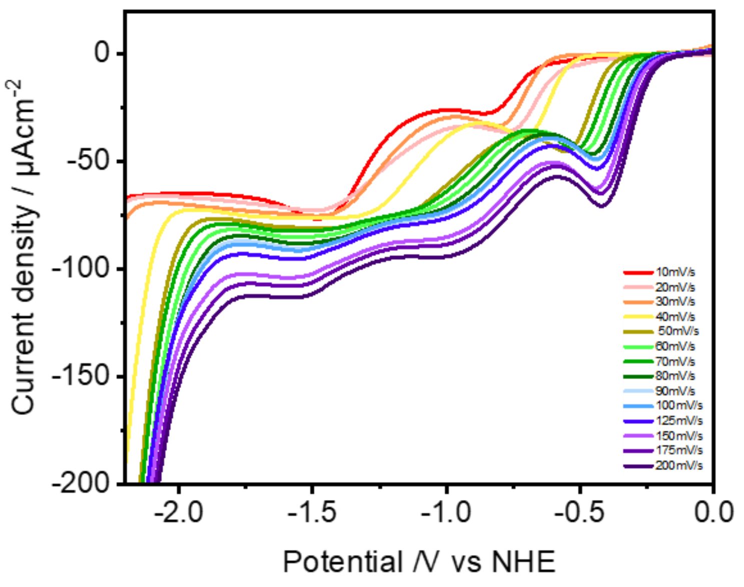

Figure S11. LSV curves of (Cl-B-SubPc) **1**dissolved in DCM under argon and CO_2 containing 0.1 M TBAP as supporting electrolyte.

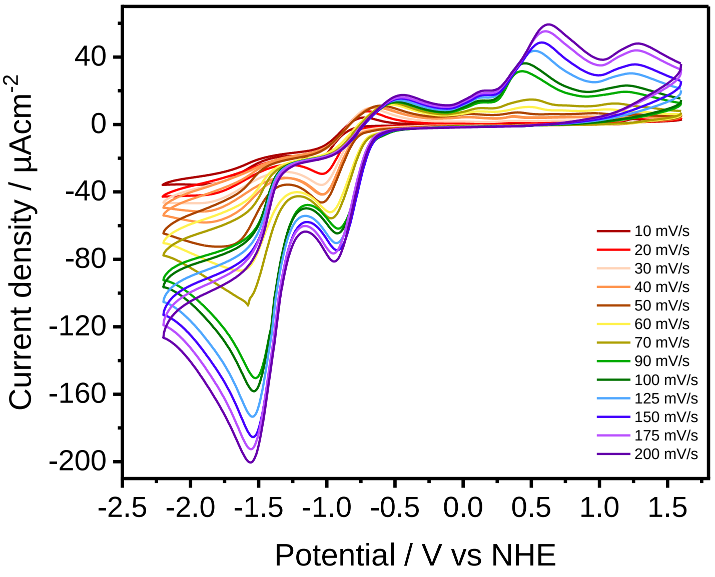

Figure S12. CV curves of (Cl-B-SubPc) **1**dissolved in DCM under argon and CO_2 containing 0.1 M TBAP as supporting electrolyte.

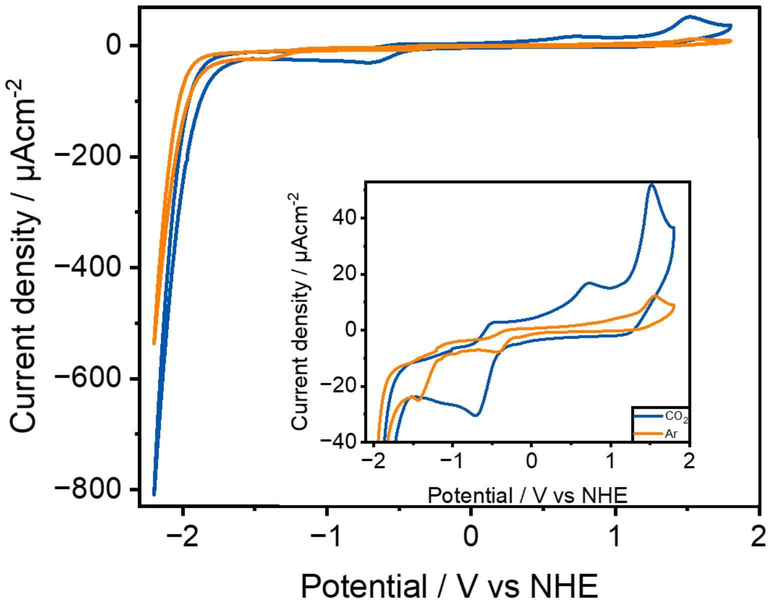

Figure S13. Comparison of cyclic voltammograms of (Cl-B-SubPc-$OC_{12}H_{23}$) **2** dissolved in DCM under argon and CO_2, containing 0.1 M TBAP as supporting electrolyte with glassy carbon as working, platinum wire as counter, and nonaqueous pseudo-Ag/AgCl as reference electrode with a scan rate of 30 mVs^{-1}.

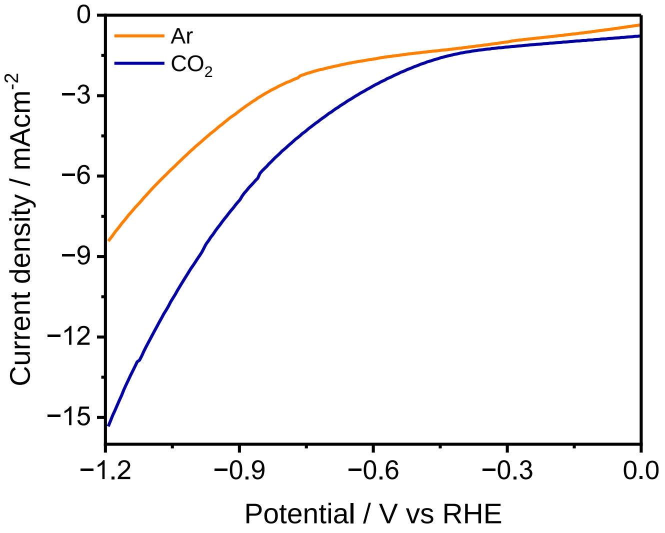

Figure S14. LSV curves at a scan rate of 30 mV s^{-1} (Cl-B-SubPc) **1**/CB WEs with Ar and CO_2 saturation 0.1M $CsHCO_3$

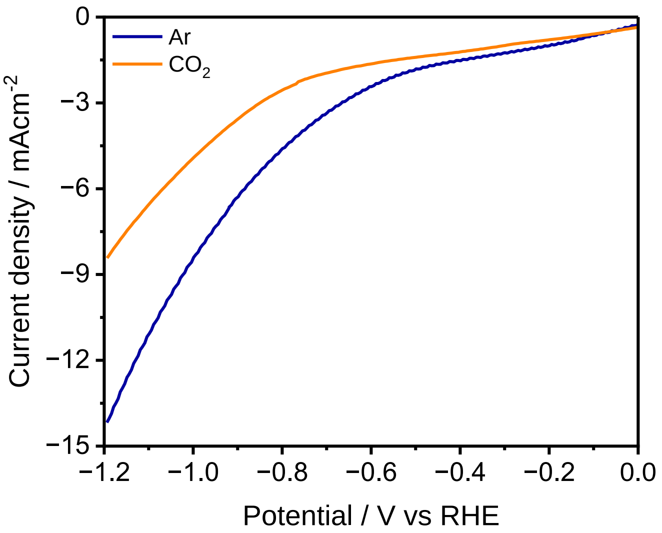

Figure S15. LSV curves at a scan rate of 30 mV s^{-1} (Cl-B-SubPc-$OC_{12}H_{23}$) **2**/CB WEs with Ar and CO_2 saturation 0.1M $CsHCO_3$

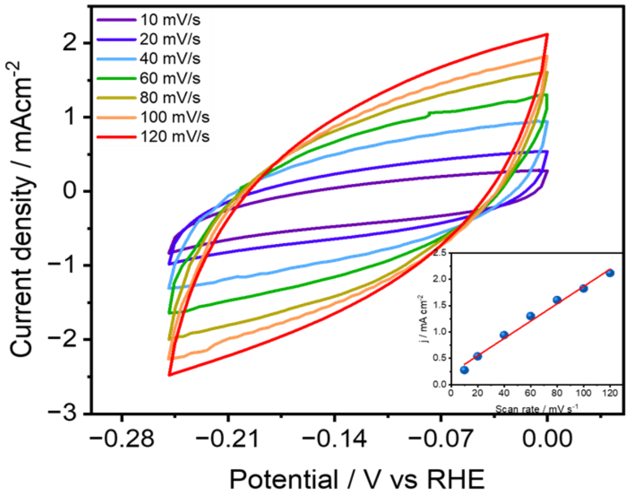

Figure S16. Cyclic voltammograms of (Cl-B-SubPc) **1** at different sweep rates of 10, 20, 40, 60, 80, 100, and 120 mV s^{-1} in 0.1M $CsHCO_3$ (b) a linear plot of capacitive current versus scan rate.

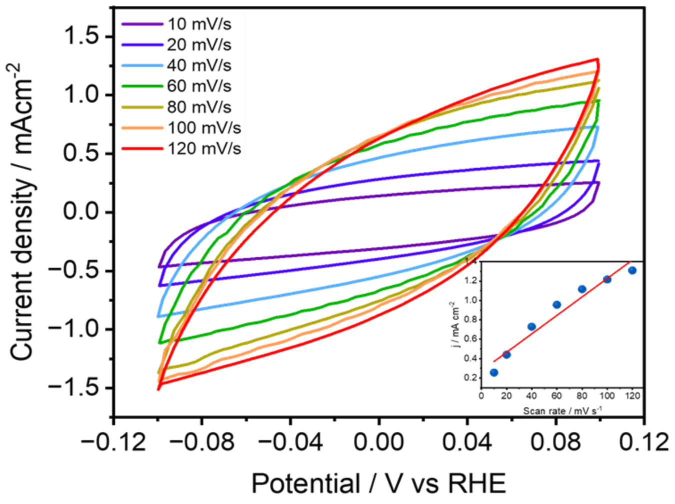

Figure S17. Cyclic voltammograms of (Cl-B-SubPc-$OC_{12}H_{23}$) **2** at different sweep rates of 10, 20, 40, 60, 80, 100, and 120 mV s^{-1} in 0.1M $CsHCO_3$ (b) a linear plot of capacitive current versus scan rate.

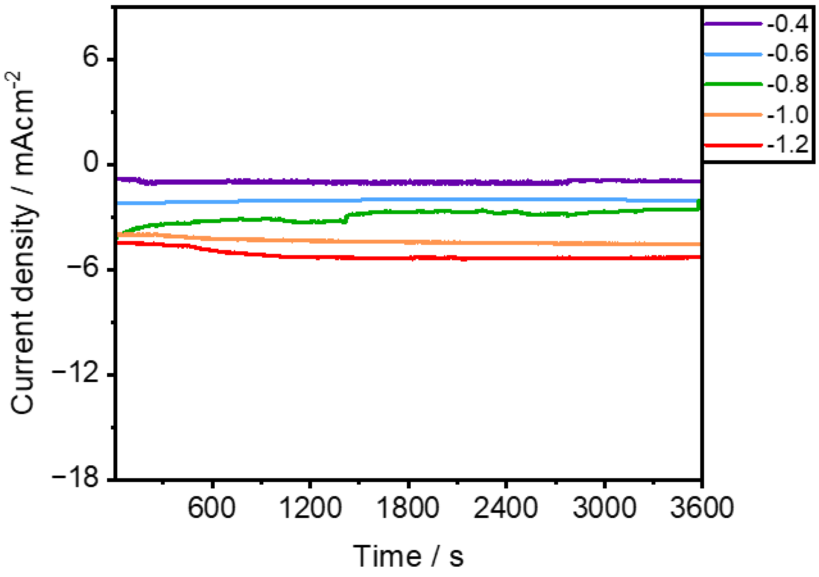

Figure S18. Cell current vs. time plot of (Cl-B-SubPc) **1** at different half-cell potentials vs. RHE.

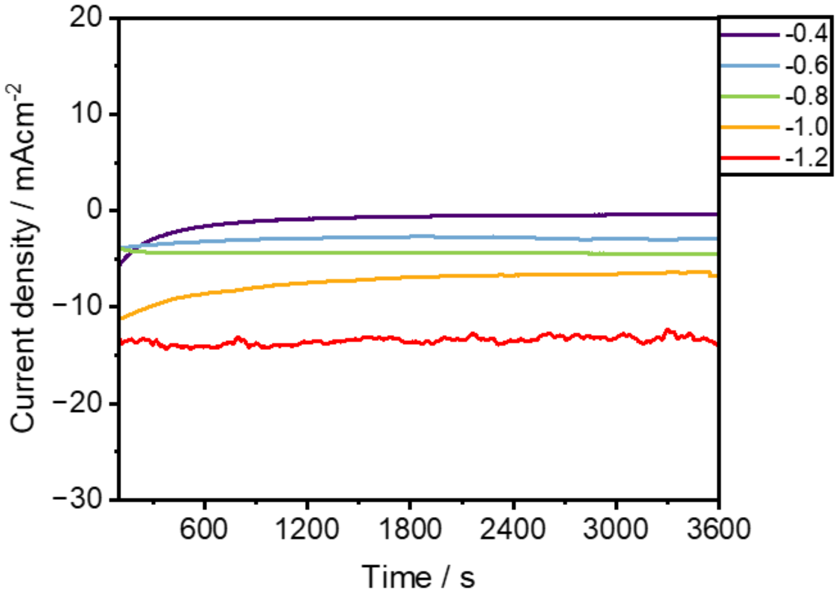

Figure S19. Cell current vs. time plot of (Cl-B-SubPc-$OC_{12}H_{23}$) **2** at different half-cell potentials vs. RHE.

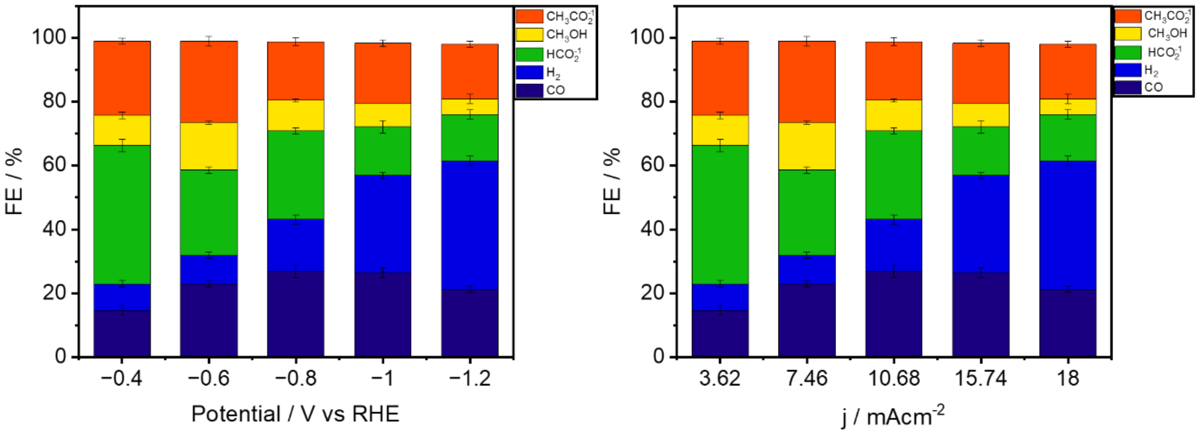

Figure S20. A) Faradaic efficiencies for CO, H_2, HCO_2^- , CH_3OH, and $CH_3CO^{-1}{}_2$ obtained during one hour electrolysis at each potential displayed. B) Faradaic efficiencies for CO, H_2, $HCOO^-$, CH_3OH, and CH_3COO^- obtained during one-hour electrolysis at each current density displayed. (Cl-B-SubPc) **1**/CB.

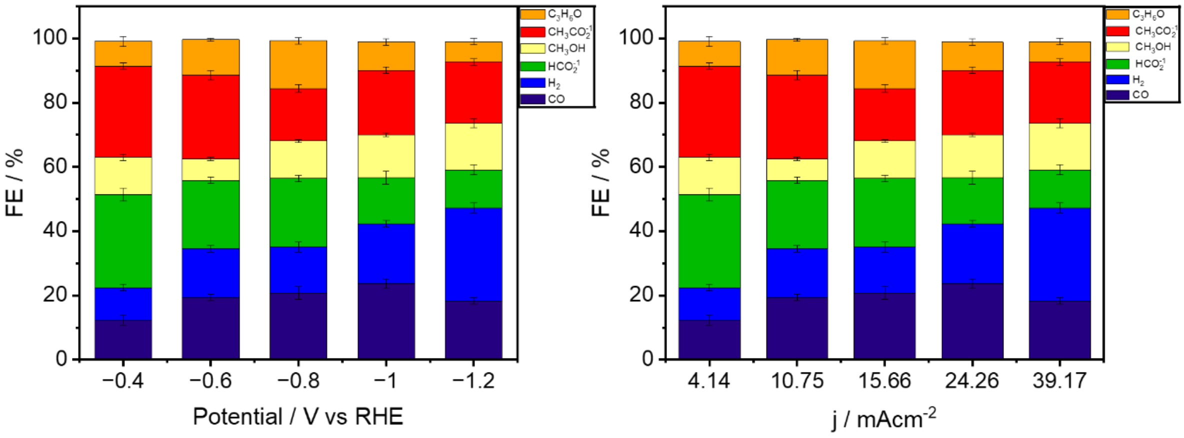

Figure S21. Faradaic efficiencies for CO, H_2, HCO_2^- , CH_3OH, $CH_3CO^{-1}{}_2$, and C_3H_6O obtained during one hour electrolysis at each potential displayed. B) Faradaic efficiencies for CO, H_2, $HCOO^-$, CH_3OH, CH_3COO^-, and acetone obtained during one-hour electrolysis at each current density displayed. (Cl-B-SubPc-$OC_{12}H_{23}$) **2**/CB

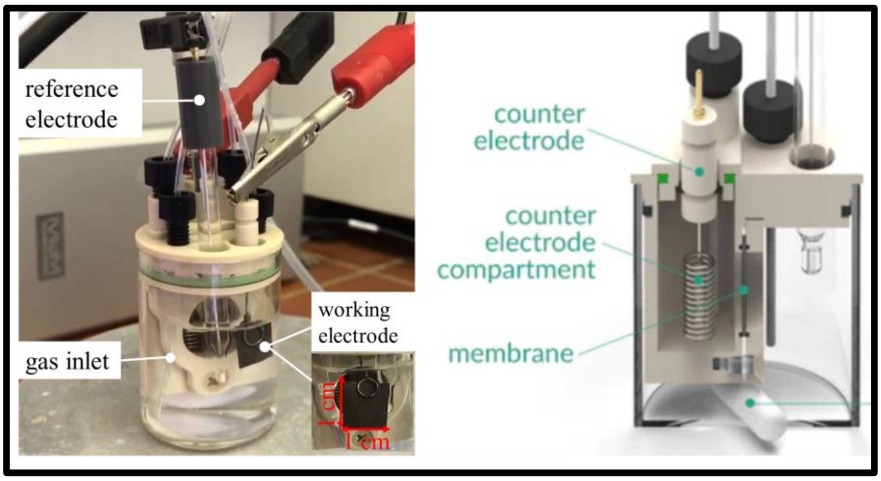

Figure S22. The electrolysis H-cell.

5. Characterization by Electrochemical Impedance Spectroscopy (EIS)- Method

The electrochemical impedance spectroscopy (EIS) was measured using an IVIUM CompactStat (Netherlands). The impedance spectrum was recorded for all experiments in the frequency range of 10^5 Hz to 0.01 Hz with a perturbation amplitude of 10 mV. The aim of this characterization is the investigation of Boron subphthalocyanines complex for the carbon dioxide reduction electrolysis, first two platinum electrodes were measured in a one-cell compartment with the corresponding electrolyte as a control experiment to determine the electrolyte resistance. Subsequently, the setup was transitioned to an H-cell configuration with a Nafion membrane, enabling the determination and subtraction of the membrane resistance from the electrolyte resistance. Further experiments involved replacing one platinum electrode with a carbon paper electrode as the working electrode. Lastly, the carbon paper, coated with (Cl-B-SubPc) ***1*** and (Cl-B-SubPc-$OC_{12}H_{23}$) **2**, served as the working electrode for the complete electrochemical cell evaluation through EIS. The resulting fitted and calculated impedance data, as well as resistance values for each cell component.

The Bode plot for the two-electrode system is also shown in (Figure S23). All resistance values for each cell component, i.e., electrolyte solution, membrane, and carrier electrode are shown in Table S1 for carbon dioxide reduction cell systems, respectively. Based on EIS, the applied electrochemical cells were characterized in detail indicating negligible losses of the systems.

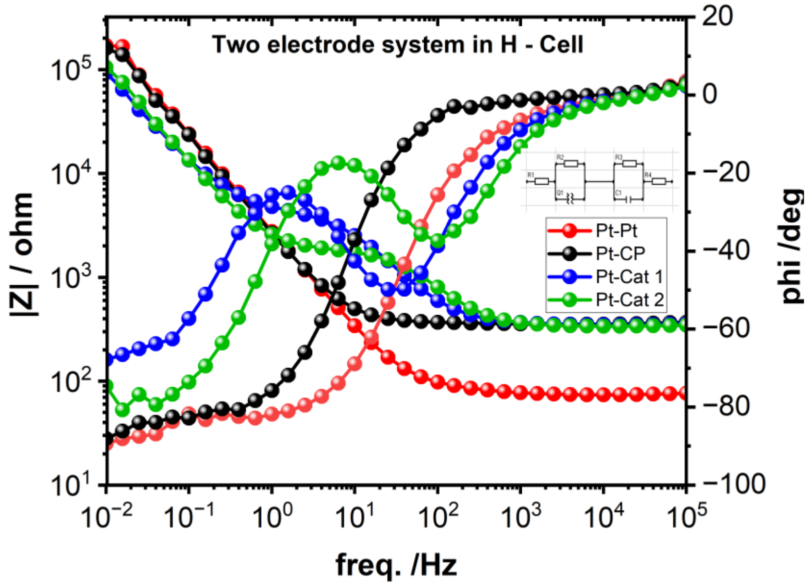

Figure S23. Bode plot recorded via electrochemical impedance spectroscopy of (Cl-B-SubPc) **1**/CB and (Cl-B-SubPc-$OC_{12}H_{23}$) **2**/CB catalyst modified carbon paper electrode in the frequency range of $1*10^{-1}$ Hz to $1*10^{5}$ Hz with a perturbation amplitude of 10 mV.

Table S 1 Cell parameters extracted via electrochemical impedance measurements.

WE	CE	R_{sol}/ Ω	$R_{carrier}$/ Ω	R/ Cat Ω	R_{Me} / Ω	C_{cat} /F	CPE-T	CPE-p
Pt	Pt	5.2E+01	4.8E+04	-	3.1E+02	-	9.1E-05	8.8E-01
GC	Pt	5.2E+01	4.5E+04	-	3.1E+02	-	5.2 E-05	8.9E-01
Cat 1	Pt	5.2E+01	2.3E+04	2.3E+02	3.1E+02	4.2E-06	2.1 E-05	8.7E-01
Cat 2	Pt	5.2E+01	1.2E+04	3.4E+02	3.1E+02	3.6E-06	1.9 E-05	8.5E-01

6. Product analysis by GC-BID

Product analysis, conducted via Gas-Chromatograph Nexis GC-2030 by Shimadzu. During the electrolysis in the closed cell, 250 µL of the headspace gas was taken for the quantification of evolved gas. CO and H_2 were detected during the electrolysis in the cathodic region. For quantification purposes, the peak area was converted into a gas volume using the calibration curve.

The Faradaic efficiency (FE%) of the gas products can be quantified following this formula,

$$FE_i = \frac{z_i \cdot n_i \cdot F}{Q_{\text{total}}} = \frac{z_i \cdot \frac{f_{CO_2} \cdot t}{V_{\text{loop}}} \cdot x_i}{Q_{\text{total}}} \cdot F$$

where z_i, n_i, Q_{total}, f_{CO2}, t, V_{loop}, x_i, and F are the number of electrons involved in the reaction, the mole amount of the total product (mol), the total consumed charge (C), the flow rate of CO_2 (5 mL min^{-1}), the test time (60 min), the volume of the quantitative loop in GC instrument used for detection (0.25 mL), the mole amount of the product directly measured by GC instrument (mol) and the faradic constant (96485.33 C mol^{-1}), respectively.

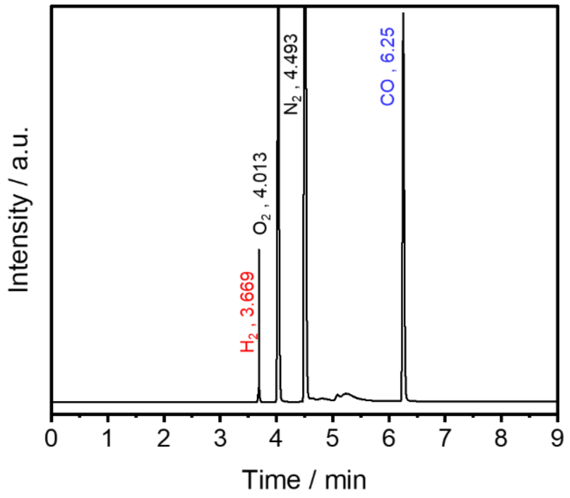

Figure S24. GC-BID-chromatogram gas products formed after CO_2 reduction at -0.6 V vs RHE for 1 h by (Cl-B-SubPc) **1**/CB catalyst modified carbon paper electrode in 0.1 M CO_2-saturated $CsHCO_3$ solution.

7. Product detection and quantification by ^{1}H-NMR

All liquid products have been quantified by a Bruker Avance III 300 MHz NMR spectrometer and a Bruker Avance III 500 MHz NMR spectrometer using D_2O as solvent. 50 µL of DMSO solution was used as internal standard. For this purpose, we have followed a previously reported procedure.[10] 450 µL analyte was taken directly from the reaction mixture for the ^{1}H-NMR analysis. Further suppression of the water peak was conducted in order to make the CO_2-reduced product peaks visible. Formate was found as only reduced product from ^{1}H-NMR with corresponding peaks at 8.2 ppm respectively (Figure S26). Number of scans and other spectral acquisition parameters were kept fixed during all acquisitions. During the quantification of the samples, each peak was normalized with respect to the DMSO peak at 2.7 ppm.

The Faradaic efficiency (*FE*, %) of the liquid products can be quantified according to this formula,

$$FE_i = \frac{z_i \cdot n_i \cdot F}{Q_{\text{total}}} = \frac{z_i \cdot \frac{V}{V_{\text{test}}} \cdot y_i}{Q_{\text{total}}} \cdot F$$

where y_i, V and V_{test} are the mole amount of liquid product directly measured by ^{1}H NMR spectra, the volume of total electrolyte (30 mL) and the volume of the tested electrolyte (0.2 mL), respectively.

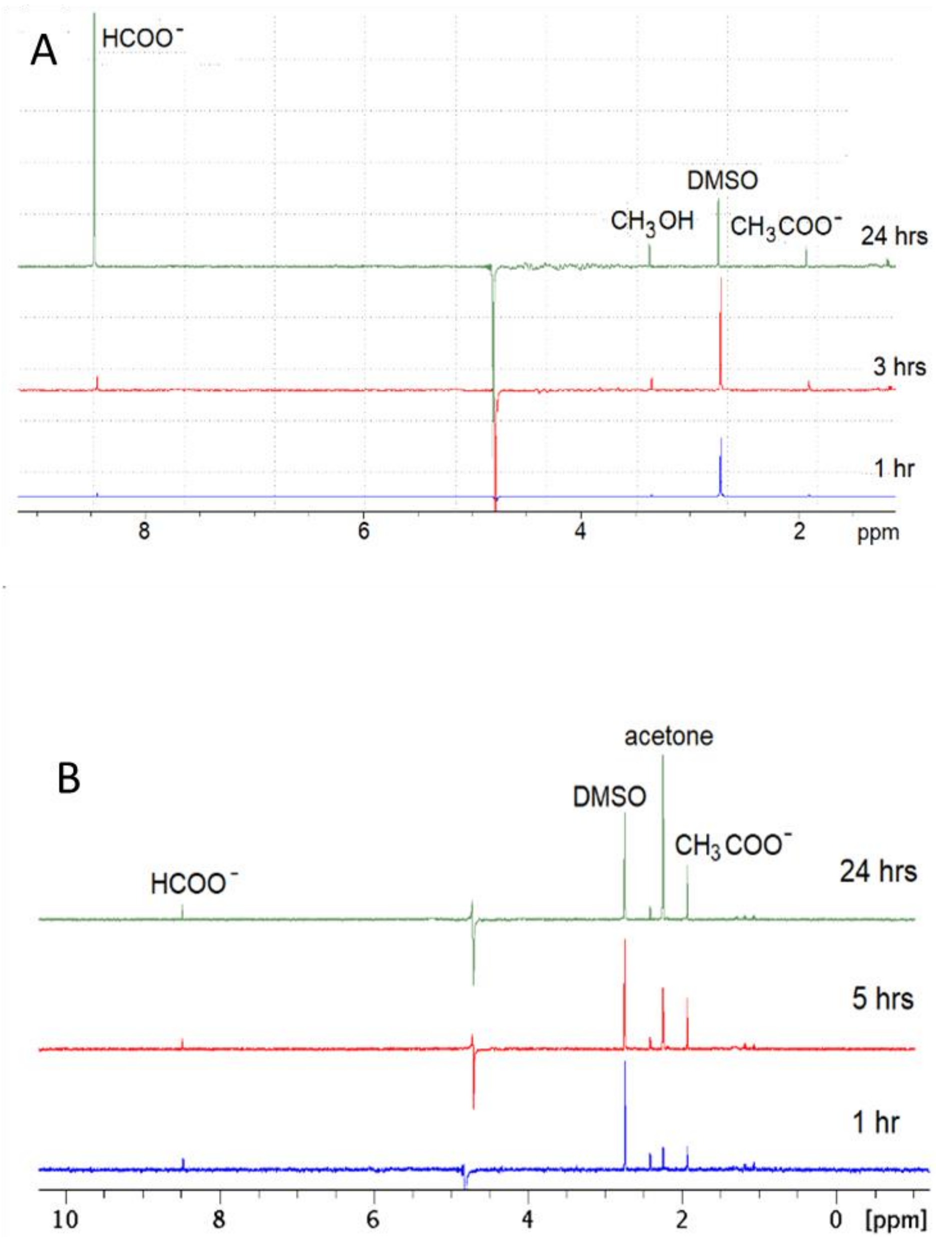

Figure S25. A) ^{1}H NMR spectrum showing the formation of formate (δ = 8.44 ppm), methanol (δ = 3.33 ppm), and acetate (δ = 1.93 ppm) after 24 hours of electrocatalysis with (Cl-B-SubPc) ***1*** at -0.8 V vs. RHE. B) ^{1}H NMR spectrum revealing the formation of formate (δ = 8.44 ppm), acetone (δ = 2.24 ppm), and acetate (δ = 1.93 ppm) under the same electrocatalytic conditions with (Cl-B-SubPc-$OC_{12}H_{23}$) **2.** DMSO is used as reference (δ = 2.74 ppm).

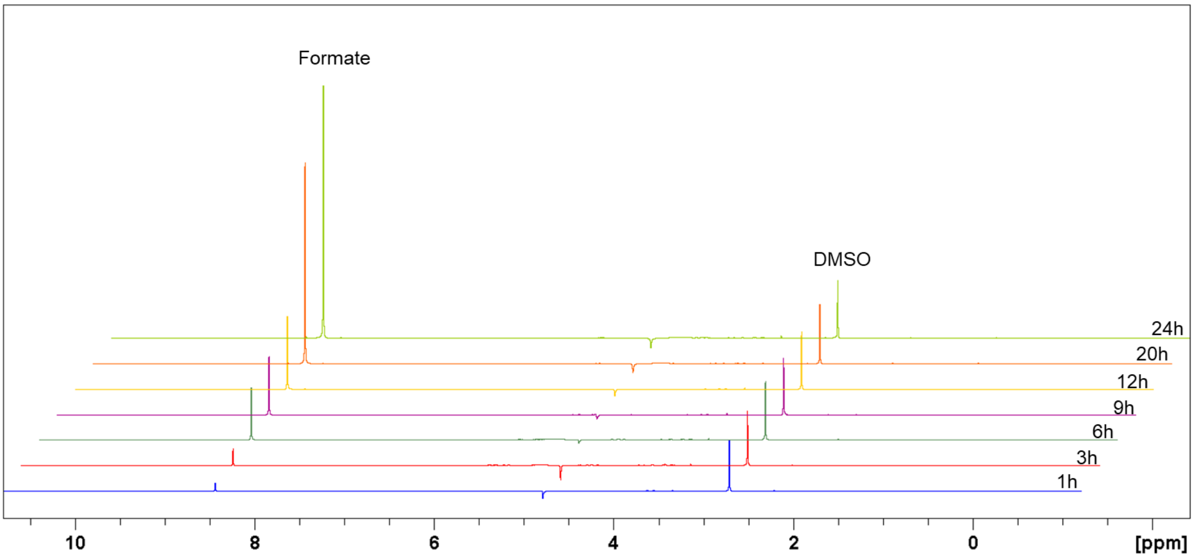

Figure S26. Time-dependent ^{1}H-NMR spectra of the liquid products formed after CO_2 reduction at -0.4 V vs RHE by (Cl-B-SubPc) **1**/CB catalyst-modified efficiency Carbon Paper electrode in 0.1M CO_2 -saturated $CsHCO_3$ solution.

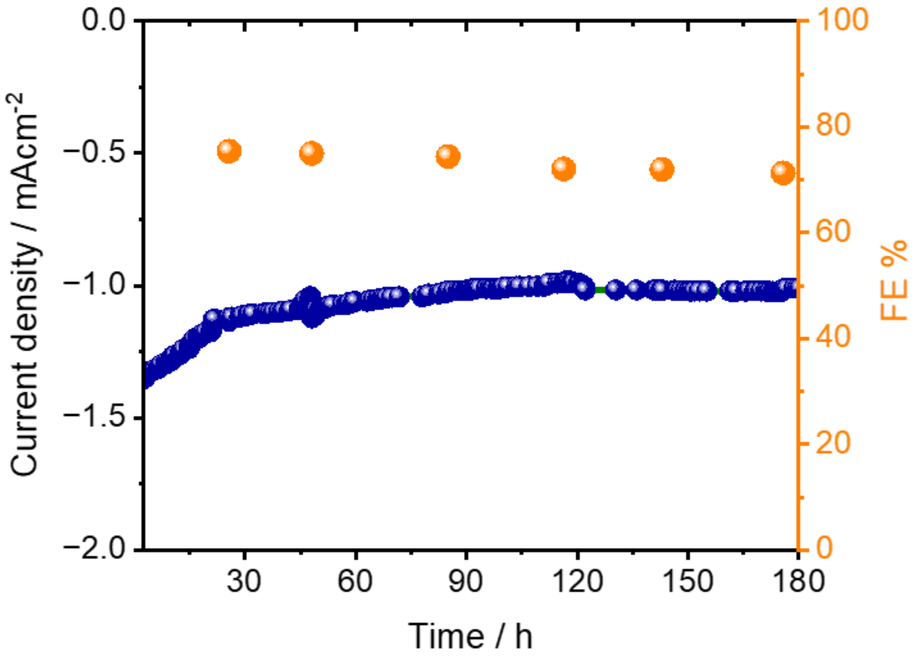

Figure S27. Long-term electrolysis experiment of the (Cl-B-SubPc) **1** /CB at -0.4 V vs. RHE of total liquid products.

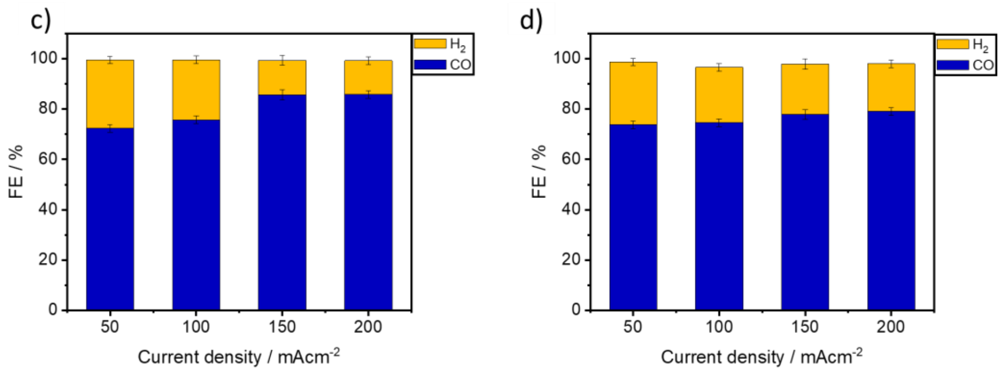

Figure S28. a) Performance of (Cl-B-SubPc) **1** for the CO_2 reduction at 50, 100, 150, and 200 mA cm^{-2} at 60 °C after two hours of electrolysis. All investigated GDEs possessed a catalytic loading of 0.5 mg cm^{-2} of active material. b) Performance of (Cl-B-SubPc-$OC_{12}H_{23}$) **2** for the CO_2 reduction at 50, 100, 150, and 200 mA cm^{-2} at 60 °C after two hours of electrolysis. All investigated GDEs possessed a catalytic loading of 0.5 mg cm^{-2} of active material.

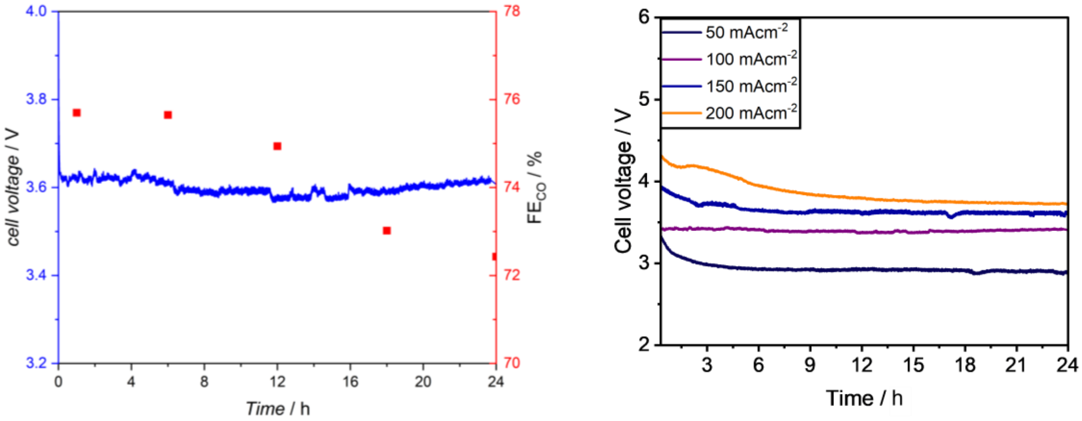

Figure S29. Long-term electrolysis experiment of the (Cl-B-SubPc) 1/CB in the ZGC at 100 mA cm^{-2}. Cell voltage vs. time plot for electrolysis experiments of 1 mg cm^{-2} Cl-B-SubPc 1/CB in the ZGC at 100 mA cm^{-2}.

Table S1: e-CO_2RR product yield of (Cl-B-SubPc) **1** at –0.4 V vs RHE relative to consumed carbon dioxide.

Product	FE (%)	e^- per mol	CO_2 / mol product	Type	CO_2 usage % = FE / e^- × CO_2	Relative Yield (%)
CO	14.57	2	1	C_1	7.284%	20.02
Formate	43.45	2	1	C_1	21.7256%	59.73
Methanol	9.41	6	1	C_1	1.568%	4.31
Acetate	23.19	8	2	C_2	5.7975%	15.94

Table S2: e-CO_2RR product yield of (Cl-B-SubPc) **1** at –0.8 V vs RHE relative to consumed carbon dioxide.

Product	FE (%)	e^- per mol	CO_2 / mol product	Type	CO_2 usage % = FE / e^- × CO_2	Relative Yield (%)
CO	26.92	2	1	C_1	13.42	39.99
Formate	27.74	2	1	C_1	13.87	41.60
Methanol	9.49	6	1	C_1	1.58	4.73
Acetate	18.25	8	2	C_2	4.56	13.68

Table S3: e-CO_2RR product yield of (Cl-B-SubPc-$OC_{12}H_{23}$) **2** at –0.4 V vs RHE relative to consumed carbon dioxide.

Product	FE (%)	e^- per mol	CO_2 / mol product	Type	CO_2 usage % = FE / e^- × CO_2	Relative Yield (%)
CO	8.24	2	1	C_1	4.12	14.42
Formate	28.17	2	1	C_1	14.09	49.31
Methanol	11.67	6	1	C_1	1.95	6.81
Acetate	28.06	8	2	C_2	7.02	24.56
Acetone	7.46	16	3	C_3	1.40	4.90

Table S2: e-CO_2RR product yield of (Cl-B-SubPc-$OC_{12}H_{23}$) **2** at –0.8 V vs RHE relative to consumed carbon dioxide.

Product	FE (%)	e^- per mol	CO_2 / mol product	Type	CO_2 usage % = FE / e^- × CO_2	Relative Yield (%)
CO	20.07	2	1	C_1	10.04	34.59
Formate	21.68	2	1	C_1	10.84	37.36
Methanol	8.03	6	1	C_1	1.34	4.61
Acetate	16.20	8	2	C_2	4.05	13.96
Acetone	14.67	16	3	C_3	2.75	9.48

TON TOF calculations

Turn Over Frequency Calculations:

Turn Over Frequency (TOF) was calculated using the equation;

$$\text{TOF} = \frac{iE_F}{NFn_{cat}}$$

Where i = current

E_F = Faradaic efficiency for ethanol

N = Number of electrons in the half-reaction (N = 2 for CO_2 to CO conversion)

F = Faraday constant

n_{cat} = total moles of the catalyst employed for the electrolysis

Turn Over Number Calculations:

Turn Over Number was calculated using the equation;

$$\text{TON} = \text{TOF} \times t$$

8. Spectroelectrochemistry and chemical reduction experiments

The electrochemical redox behavior of Boron subphthalocyanine chlorides was studied under Ar and CO_2 atmosphere using spectroelectrochemical UV/vis (SEC-UV/vis) experiments. These experiments were conducted in an optical thin layer electrode cell (OTTLE cell), where a light transparent platinum mini-grid acted as working, as counter and a Ag-microwire as the pseudo-reference electrode. Experiments were pre-treated for 1 min at constant potential, with subsequent start of the UV/vis acquisition and electrolysis at identical conditions [3].

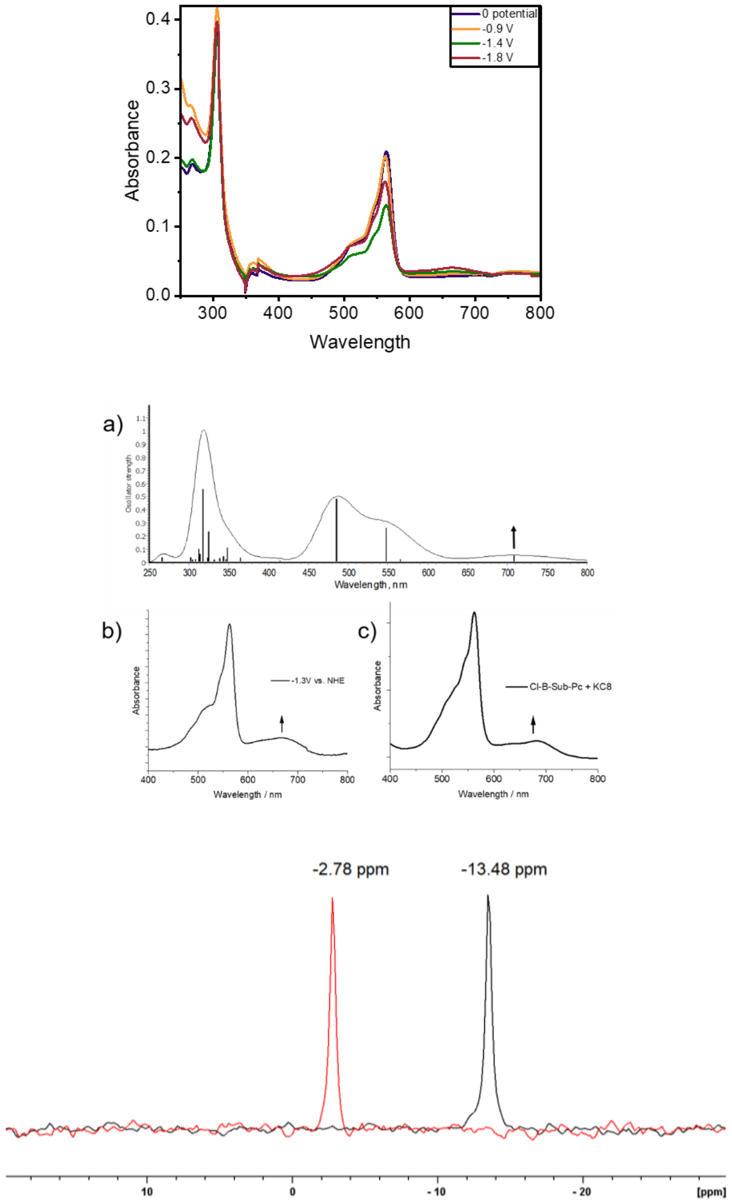

Figure S30. UV-vis spectroelectrochemistry of (Cl-B-SubPc) ***1*** in 0.2 M TBAP (in dry DCM), at potentials from -0.5 to -1.7 V vs Ag/AgCl. Kinks at 350 nm stem from lamp changing of the UV-vis spectrometer. Computed TD-DFT UV-vis spectrum of B-SubPc anion radical and b) UV Vis spectrum at -1.3 V vs NHE and c) UV vis spectrum after chemical reduction with KC8. ^{11}B-NMR of with KC_8 chemically reduced (Cl-B-SubPc) ***1*** (500 MHz, THF-d_8, 25 °C).

For the chemical reduction tests an excess of KC_8 was added to (Cl-B-SubPc) ***1*** in an argon filled glovebox and then dissolved in dry acetonitrile for UV-vis spectroscopy. Afterwards a drop of water was added to this mixture via syringe. For the ^{1}H-NMR measurement aforementioned reactants were dissolved in non-dried THF.

9. Geometry Optimization, Mechanistic Insights

The carbon monoxide formation proceeds through the initial one-electron reduction of Cl-B-SubPc catalyst, forming again the highly reactive anion radical: B-SubPc + e^- → $[B\text{-}SubPc]^{\bullet -}$. The reduced $[B\text{-}SubPc]^{\bullet -}$ species interacts with CO_2 through nucleophilic attack, leading to the formation of a bent CO_2 adduct: $[B\text{-}SubPc]^{\bullet -}$+$CO_2$ → $CO_2^{\bullet -}$−B-SubPc. Subsequent PCET leads to the CO-H_2O-BsubPC intermediate splits of the CO and H_2O from the B-SubPC complex. The methanol formation proceeds through the initial one-electron reduction of Cl-B-SubPc catalyst, forming again the highly reactive anion radical: B-SubPc + e− → **[B-SubPc]•−.** The reduced [B-SubPc]•− species interacts with CO_2 through nucleophilic attack, leading to the formation of a bent CO_2 adduct: $[B\text{-}SubPc]^{\bullet -}$+$CO_2$ → $CO_2^{\bullet -}$−B-SubPc**.** Subsequent PCET leads to the CO-H_2O-BsubPC. Instead of splitting off the CO and H_2O from the B-SubPC complex, multiple PCETs produce the HCO-B-SubPc intermediate and finally the methanol, which can be observed at 3.32 ppm in the ^{1}H NMR spectra of the electrolyte solution after electrocatalysis experiments. The catalytic cycle is completed by the regeneration of the active [B-SubPc]•− species, ensuring sustained CO_2 reduction over extended electrolysis periods. The electrocatalytic pathway leading to acetate formation proceeds through a series of well-defined PCET steps. The first step involves the dimerization of surface-bound *CO species via C–C bond formation, generating the critical *OCCO intermediate. This step is a key determinant of C_2 product selectivity and is energetically favorable under low-overpotential conditions. Subsequent reductions of *OCCO proceed via stepwise protonation and electron transfer, yielding glyoxal (*CHO-CHO) as a transient intermediate. Further hydrogenation leads to the formation of hydroxyacetaldehyde (*CH_2OH-CHO), which subsequently converts into acetic acid. Under alkaline conditions, acetic acid deprotonates to form acetate, which can be observed at 1.87 ppm in the ^{1}H NMR spectra of the electrolyte solution after electrocatalysis experiments The catalytic cycle is completed by the regeneration of the active [B-SubPc]•− species, ensuring sustained CO_2 reduction over extended electrolysis periods. Surprisingly, for catalyst **2**, after electrocatalysis at -0.8 V - -1 V vs RHE, acetone production is observed. ^{1}H NMR spectra in Figure 6e exhibits an increasing ^{1}H NMR resonance at 2.24 ppm. In addition, a small dublet signal is observed at 1.1 ppm reflecting 2-propanol as the reduction product of acetone. The proposed reaction path towards acetone follows the sequence is assumed to be similar to the reported one by Chen et al. and Koper et al.: CO_2 → COOH* →

CO* → COCO* → COCOH* → COC* → COCH* → $COCH_2$* → $COCH_3$*→$COCOCH_3$* → $COHCOCH_3$* → $CCOCH_3$* → $CHCOCH_3$* → CH_2COCH_3* → CH_3COCH_3 → CH_3-CHOH-CH_3.

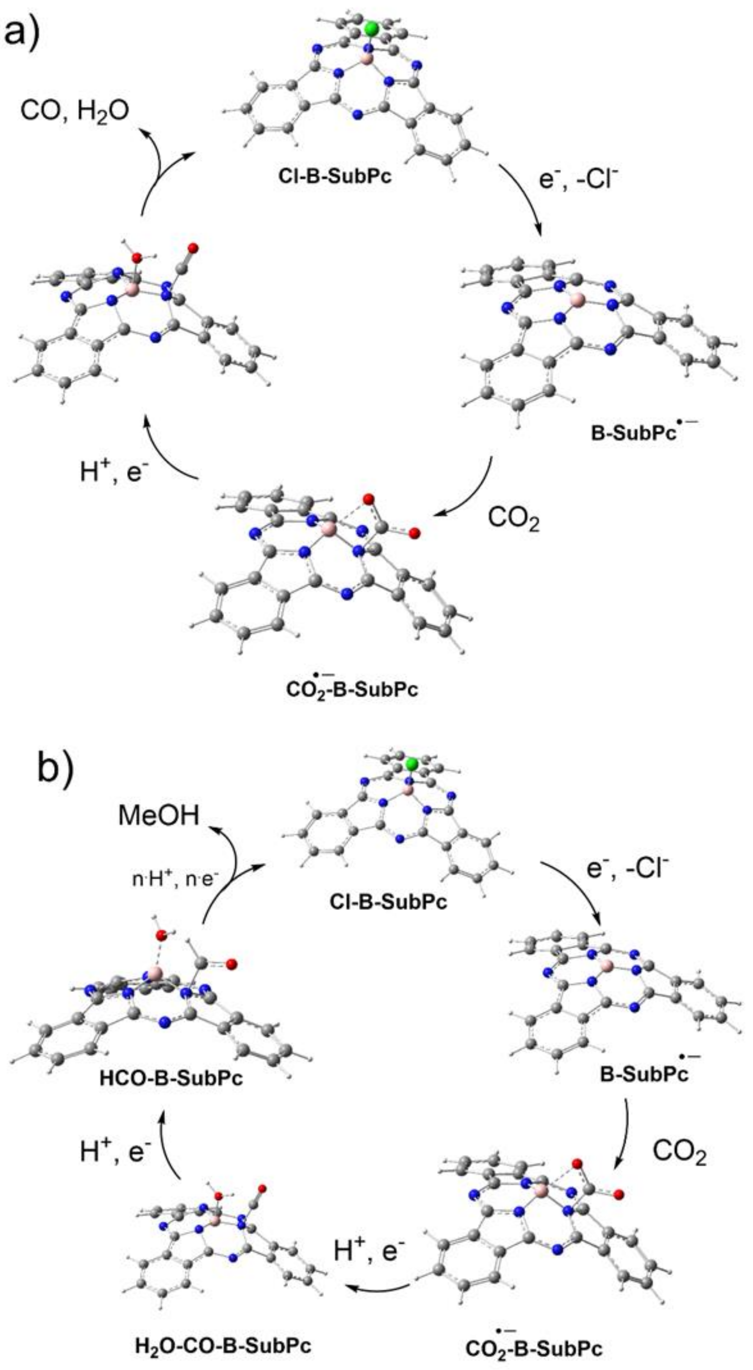

Figure S31. Proposed mechanisms for a) CO and b) methanol production.

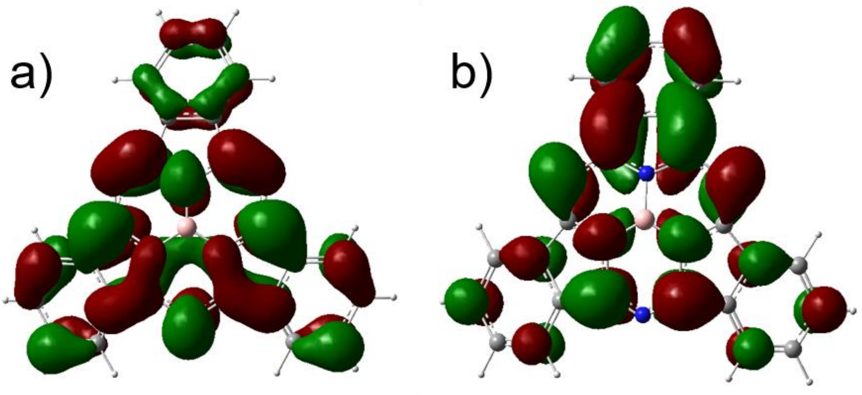

Figure S32. HOMO and LUMO of B-SubPc anion radical.

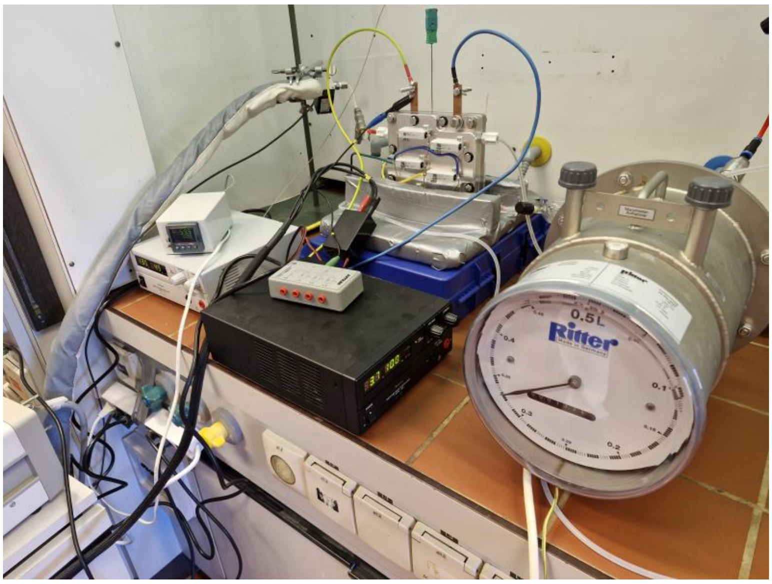

Figure S33. Lab-scale electrolyzer equipment.

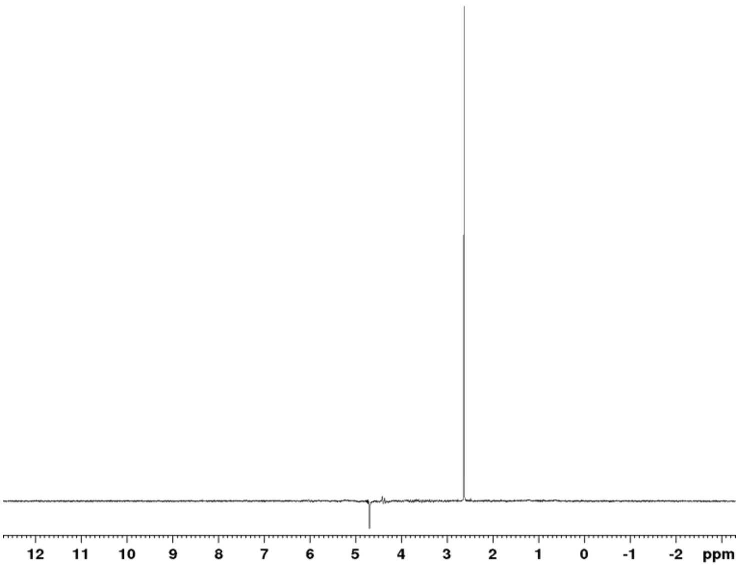

Figure S34. ^{1}H NMR spectrum Control electrolysis performed in an Ar-saturated electrolyte**.**

10. References

[1] Swarts, P. J.; Conradie, J. Electrochemical behaviour of chloro- and hydroxy-subphthalocyanines, Electrochimica Acta, Volume 329, 2020, 135165, ISSN 0013-4686.

[2] Coskun, H.; Aljabour, A.; Luna, P. de; Farka, D.; Greunz, T.; Stifter, D.; Kus, M.; Zheng, X.; Liu, M.; Hassel, A. W.; Schöfberger, W.; Sargent, E. H.; Sariciftci, N. S.; Stadler, P. Biofunctionalized conductive polymers enable efficient CO_2 electroreduction. *Sci. Adv.* **2017**, *3* (8), 68.

[3] Gonglach, S.; Paul, S.; Haas, M.; Pillwein, F.; Sreejith, S. S.; Barman, S.; De, R.; Müllegger, S.; Gerschel, P.; Apfel, U.-P.; Coskun, H.; Aljabour, A.; Stadler, P.; Schöfberger, W.; Roy, S. Molecular cobalt corrole complex for the heterogeneous electrocatalytic reduction of carbon dioxide. *Nature communications* **2019**, *10* (1), 3864.

[4] Pellumbi, K.; Krisch, D.; Rettenmaier, C.; Awada, H.; Sun, H.; Song, L.; Sanden, S. A.; Hoof, L.; Messing, L.; Puring, K. j.; Siegmund, D.; Cuenya, B. R.; Schöfberger, W.; Apfel, U.-P. Pushing the Ag-loading of CO_2 electrolyzers to the minimum via molecularly tuned environments. *Cell Reports Physical Science* **2023**, *4* (12), 101746.
